# Inter-nucleosomal communication between histone modifications for nucleosome phasing

**DOI:** 10.1371/journal.pcbi.1006416

**Published:** 2018-09-06

**Authors:** Weizhong Chen, Yi Liu, Shanshan Zhu, Guoyu Chen, Jing-Dong J. Han

**Affiliations:** 1 Key Laboratory of Computational Biology, CAS Center for Excellence in Molecular Cell Science, Collaborative Innovation Center for Genetics and Developmental Biology, Chinese Academy of Sciences-Max Planck Partner Institute for Computational Biology, Shanghai Institutes for Biological Sciences, University of Chinese Academy of Sciences, Chinese Academy of Sciences, Shanghai, China; 2 Beijing Key Lab of Traffic Data Analysis and Mining, School of Computer and Information Technology, Beijing Jiaotong University, Beijing, China; UNITED STATES

## Abstract

Combinatorial effects of epigenetic modifications on transcription activity have been proposed as “histone codes”. However, it is unclear whether there also exist inter-nucleosomal communications among epigenetic modifications at single nucleosome level, and if so, what functional roles they play. Meanwhile, how clear nucleosome patterns, such as nucleosome phasing and depletion, are formed at functional regions remains an intriguing enigma. To address these questions, we developed a Bayesian network model for interactions among different histone modifications across neighboring nucleosomes, based on the framework of dynamic Bayesian network (DBN). From this model, we found that robust inter-nucleosomal interactions exist around transcription start site (TSS), transcription termination sites (TTS) or around CTCF binding sites; and these inter-nucleosomal interactions are often involved in transcription regulation. In addition to these general principles, DBN also uncovered a novel specific epigenetic interaction between H2A.Z and H4K20me1 on neighboring nucleosomes, involved in nucleosome free region (NFR) and nucleosome phasing establishment or maintenance. The level of negative correlation between neighboring H2A.Z and H4K20me1 strongly correlate with the size of NFR and the strength of nucleosome phasing around TSS. Our study revealed inter-nucleosomal communications as important players in signal propagation, chromatin remodeling and transcription regulation.

## Author summary

Nucleosomes are the basic unit of chromatin organization. At a global level, they fold up to form chromatin fibers in higher order structure to control the activation/repression states of chromatins. At a local level, especially around transcriptional starting sites (TSSs), nucleosomes play an important role in regulating gene expression by dynamically positioning to affect the recruitment of RNA polymerase II and transcriptional factors. In particular around actively transcribed TSSs, nucleosomes are regularly positioned to form a typical pattern of nucleosome phasing. As it suggests that the forming of nucleosome phasing is a synergistic behavior across the nucleosomes around TSS, we hypothesize that there exist communications, which is probably some propagations of histone modifications, between neighboring nucleosomes, as nucleosome functions are essentially due to histone modifications. Here, to address the question, we investigated the correlations of histone modifications across neighboring nucleosomes, and revealed a negative correlation between H2A.Z and H4K20me1 across neighboring nucleosomes. It is a development to the well accepted knowledge that H2A.Z and H4K20me1 are positively correlated at genome-wide level. In addition, we revealed a probable contribution of H2A.Z-H4K20me1 anti-correlation in nucleosome phasing around active TSSs, therefore, shedding light on understanding the forming of nucleosome phasing.

## Introduction

Epigenetic factors, such as histone modifications, are a class of important regulators of eukaryotic gene expression. In a previous study, we successfully applied a Bayesian network approach to decipher the complex histone codes for gene regulation [1]. Although the model explains the causal relations between different histone modifications at the transcription start site (TSS) and gene expression in general, that is, how different chromatin modification states affect each other and then collectively influence gene expression, an important question is still unanswered: how does this epigenetic information transfer from the TSS towards different nucleosomes to facilitate the transcriptional process?

A nucleosome is composed of an octamer, two copies of the four basic histone proteins (H2A, H2B, H3 and H4), which is wrapped around 1.67 times by a 147bp DNA. In the initial step of gene transcription, the nucleosome in the nucleosome free region (NFR) is first evicted [2]. Then, the transcription factor (TF) binding sites are exposed to facilitate the recruitment and assembly of the RNA polymerase II (Pol II) complex [3]. Several *in vitro* studies suggest that nucleosomes are in a dynamic equilibrium between a fully wrapped state and partial wrapped states [4, 5]. However, it is still unclear how the disassembly or mobility of the downstream nucleosomes is involved in the later transcription process or how histone modifications propagate across different nucleosomes. In particular, how nucleosome free region and nucleosome phasing are formed remains an enigma [6–9].

Pieces of this puzzle are starting to emerge recently. In the presence of ATP and ATP-dependent chromatin assembly and remodeling factors (ACF), such as SWI/SNF, the nucleosomes can shift along the DNA sequence gradually to balance the linker DNA length on either side of a nucleosome, and finally at equilibrium, the dimeric ACF complex continuously drives the nucleosome forward and backward [10, 11]. Particularly, different types of modifications on the tails of histone proteins affect histone-DNA interactions, the binding of regulatory proteins to nucleosomes and the nucleosome mobility [12]. For example, H2A.Z, a histone variant favorable for nucleosome shifting [13], was implied to interact with SWI/SNF family [14] to regulate nucleosome sliding synergistically [15]. Besides their contribution to the nucleosome mobility, some modifications are also shown to further recruit histone modification enzymes (e.g., HATs, HDACs or HMTs) to propagate the signals by catalyzing similar modification of neighboring nucleosomes [16]. Such interactions have been proposed to be essential for setting up a bi-stable state of chromatin domains through positive feedbacks [16, 17]. Based on these observations, we propose an inter-nucleosome histone modification regulation model, where several modifications initiated by TFs and Pol II complex, orchestrate the recruitment of certain enzymes and regulators to propagate the histone modification patterns and to facilitate the disassembly or mobility of nucleosomes, which in turn regulate transcription process.

Dynamic Bayesian network (DBN) is an extension of the classic Bayesian network model, which is composed of two graph structures: the prior network and the transition network. The former is defined for time 0 to represent *a priori* dependences and the latter represents the interdependencies between variables at consecutive frames of a time series process [18]. In the transition network, all edges are directed and they are either oriented from the previous to the current time frame or within the current time frame, to represent the Markovian assumption that the states of variables at time *t* is independent of the states of variables at time 0,1,⋯,*t*−2 given the states of variables at time *t*−1. Here, we treat two neighboring nucleosomes as two consecutive time frames and use the DBN approach to model the propagating interactions of histone modifications between neighboring nucleosomes.

Based on the inferred DBN model, we found that **(1)** Robust inter-nucleosome interactions exist in both orientations from the TSS and CTCF center to either sides, and from TTS upstream to downstream; **(2)** Inter-nucleosome interacting histone modifications inferred by the DBN model are often known to be required for transcription regulation. Moreover, we inferred and validated novel inter-nucleosomal interactions between H2A.Z and H4K20me1 at TSS in establishing/maintaining NFR and nucleosome phasing.

## Results

### Adapting DBN to infer inter-nucleosomal interactions among histone modifications and transcription factors (TFs) around TSS, TTS and CTCF-binding sites

For stationary DBN models, the edge connections are identical for any two consecutive time frames. In this case, we can conveniently represent the transition graph of a DBN as a template Bayesian network on two consecutive time frames. It then unfolds repeatedly across many time points to form a large network to represent the structure for information propagation across times. With this simplification, the problem of learning the transition network in a DBN is reduced to learning this template BN, which can be formulated as a BN-structure learning problem under specific graph constraints (Materials and methods).

**Fig 1:**
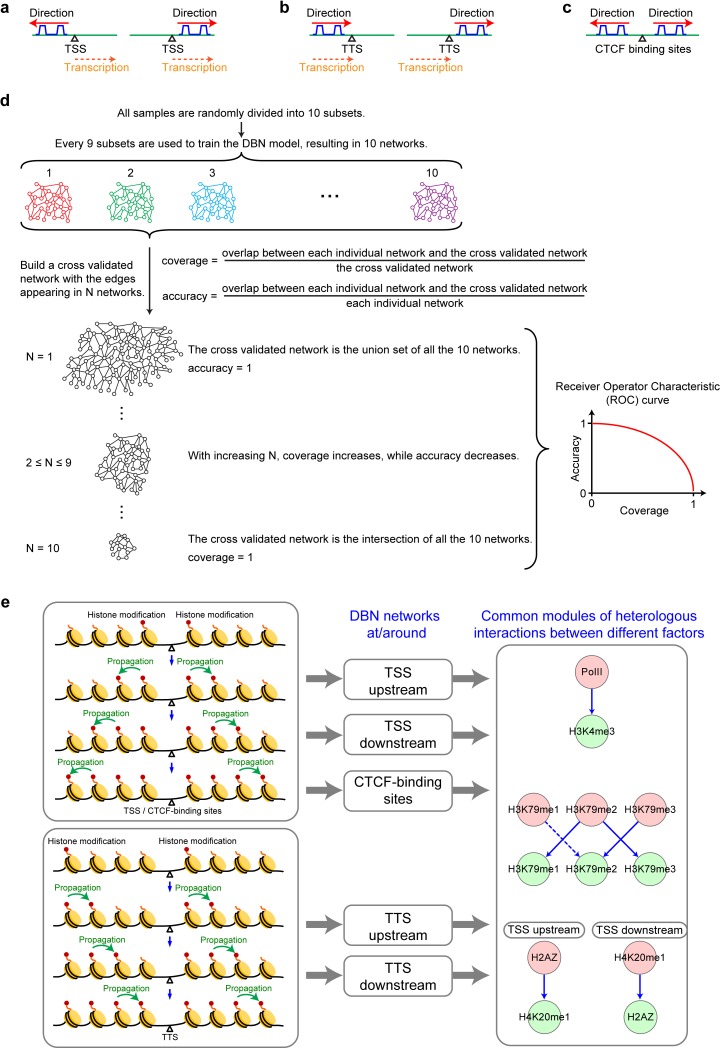
Schematic diagrams of the inter-nucleosome propagation models and three common modules in DBN inferred networks. (**a-c**) Schematic diagrams of the direction of the modeled signal propagation around TSS (a), TTS (b), and CTCF-binding regions (c). (**d**) The cross-validation scheme of dynamic Bayesian network (DBN). (**e**) Three common modules of heterologous interactions between different factors inferred by DBN at TSS, TTS and CTCF sites. The network modules depict the signal propagation from the factors at “before” nucleosomes (pink nodes) to the factors at neighboring “after” nucleosomes (green nodes).

To model the propagating interactions of histone modifications on neighboring nucleosomes, we treat two neighboring nucleosomes as two consecutive time frames in DBN. Specifically, we investigate the signal propagation model for nucleosomes around three types of chromosome regions–TSS, Transcription Termination Sites (TTS) and CTCF binding sites—based on the following considerations: **(1)** The chromatin regions around these sites carry important biological functions, such as transcription regulation or insulator function [19]. **(2)** It is more robust and reliable to perform network learning around these sites, as their surrounding nucleosomes are well-phased [13, 20–22]. **(3)** There are tens of thousands of TSS, TTS and CTCF bindings sites widely distributed in the genome, which provide sufficient samples/instances for the DBN-learning algorithm to reverse engineer the inter-nucleosome propagation principles with high confidence. **(4)** Finally, the probable directions of information flow for nucleosomes around sites can be assumed as from the center of TSS/CTCF-binding site to their upstream and downstream chromosomal regions, respectively, i.e., a nucleosome downstream of a TSS/CTCF region will causally influence its downstream flanking nucleosome, and a nucleosome upstream of the region will causally influence its upstream flanking nucleosome (Fig 1). This is because the distribution of some modifications (for example, H3K4me2, H3K4me1, H3K27me3, H3K27me1, H3K79me3, H3K9me1, H2A.Z, etc.) tends to be symmetric around the center of a TSS/CTCF site and the pattern is usually strong and clear at the center and gradually attenuates along either direction [20]. For TTS, signals are often continuous from TTS upstream to downstream, we therefore assumed the direction of information flow as from TTS upstream to downstream (Fig 1), in the transcriptional direction. In contrast, the information propagation structure is not clear for arbitrary chromosomal regions, where both the information flows from 5’ to 3’ and 3’ to 5’ are plausible for neighboring nucleosomes, leading to ambiguities in inferring the signal propagation model.

### Consensus networks of inter-nucleosomal interactions among histone modifications and transcription factors (TFs) around TSS, TTS and CTCF-binding sites

Obtaining accurate genome-wide nucleosome position information is a prerequisite to study inter-nucleosome propagation. The MNase-based sequencing (MNase-seq) data of human CD4+ T cells [13] has offered a genome-wide map of nucleosome positions in the human genome. We first run our improved algorithm for nucleosome positioning from sequencing data (iNPS) [23] to analyze the MNase-seq data [13]. Then, we further selected regularly phased nucleosomes (well-isolated “MainPeak” nucleosome with suitable peak ‘width’ of 70 ~ 90 bp and proper neighboring distance of 160 ~ 400 bp) around TSS, TTS and CTCF-binding sites (Materials and methods). Based on the calculated nucleosome positions, histone modifications and transcription factor (TFs) binding signals from a compilation of ChIP-seq data [20] were assigned to each detected nucleosome based on the genomic coordinates of the detected nucleosomes. Finally, the constraint-based DBN structure-learning algorithm is used to *de novo* infer a network model by learning from dependency of the quantities of histone modifications and TF binding at these neighboring nucleosome pairs (Materials and methods).

Table A in S1 Text lists the inter-nucleosome propagation networks obtained under different parameter settings, such as using various ranges around TSS/CTCF/TTS regions (Materials and methods) and using different “center-inclusion levels” (Materials and methods, S1A and S1B Fig) to select neighboring nucleosome pairs for DBN training. All these DBN networks are very robust as demonstrated by the Receiver Operator Characteristic (ROC) curves quantified using the cross-validation experiments (Fig 1D, see Materials and methods). All the areas under curve (AUC) values are above 0.96 (Table A in S1 Text), demonstrating the high stability of network structure against re-sampling of training data. Despite the high stability of network inference on each parameter setting, the networks inferred under different settings were not exactly the same, as expected. Nevertheless, a number of interactions have occurred in most of these networks. We extracted a final network of common interactions by overlapping most of these networks and selected the edges that have appeared in half or more of the networks (Materials and methods), which we called the “consensus networks”.

The final consensus DBN networks at TSS upstream, TSS downstream and around CTCF-binding sites characterize the inter-nucleosome propagation of histone modification and TF-binding patterns from the center of TSS or CTCF-binding region to the two flanking sides at a step-size of a single nucleosome (S1C and S1D Fig). While the consensus networks at TTS upstream and TTS downstream depict the inter-nucleosome propagation from TTS upstream to downstream (S1C and S1D Fig). We can find that the consensus networks inferred at the TSS, TTS and CTCF regions are consistent (S1E Fig), in particular most of them contain three stable common network modules: the “*Pol II–H3K4me3*” module, the “*H3K79me1*, *2*, *and 3*” module, and the “*H4K20me1 –H2A*.*Z*” module (Fig 1E).

1. The “*Pol II–H3K4me3*” module is mainly composed of the heterologous edge “Pol II → H3K4me3” between two neighboring nucleosomes. In this module, Pol II brings H3K4me3 to the TSS upstream or downstream nucleosome (Fig 1E, Table 1, and S1C Fig).

**Table 1:** The similarities and differences between the inter-nucleosome consensus network around TSS, CTCF-binding, and TTS regions.

Stable network modules of TSS, CTCF and TTS regions	“Pol II–H3K4me3” module	“H4K20me1 –H2A.Z” module	“H3K79me1, 2, and 3” module
**Basic structures**	Pol II → H3K4me3	H4K20me1 → H2A.Z	H3K79me1 ←→ H3K79me2
			H3K79me2 ←→ H3K79me3
**Specific to TSS upstream**	H3K4me1 → H3K4me3	H2A.Z → H4K20me1	
**Specific to TSS downstream**	H3K4me1 → H3K4me3	H3K4me3 → H4K20me1	H3K79me1 → H3K27me3
	H3K4me3 → H4K20me1	H3K79me3 → H2A.Z	H3K79me3 → H2A.Z
**Specific to CTCF regions**	H3K9me1 → H3K4me1, 2, 3	H3K79me1, 2, 3 → H4K20me1	H3K79me3 → H3K79me1
	H3K4me2 → H3K9me1	H4K20me1 → H3K79me1	H3K79me1, 2, 3 → H4K20me1
	H3K4me2 → H3K4me1	H4K20me1 ←→ H2BK5me1	H4K20me1 → H3K79me1
			H2BK5me1 → H3K79me1
			H3K79me1 → H3K27me3
**Specific to TTS upstream**	H2A.Z → H3K4me3	Heterologous edge missed.	H3K79me1 → H4K20me1
			H3K79me1 → me2” missed.
**Specific to TTS downstream**	H2A.Z → H3K4me3	Heterologous edge missed.	“H3K79me1 → me2” missed.

2. The “*H3K79me1*, *2*, *and 3*” module contains “two-way” interweaving edges between H3K79me1 and H3K79me2 (“H3K79me1 → H3K79me2” and “H3K79me2 → H3K79me1”) and between H3K79me2 and H3K79me3 (“H3K79me2 → H3K79me3” and “H3K79me3 → H3K79me2”). These interactions are probably due to the concentration effect and a nonprocessive methylation mechanism by Dot1, as demonstrated by Frederiks et al. [24]. Briefly speaking, at the regions far from Dot1 (which locates close to TSSs), propagation between H3K79me1 and H3K79me2 probably occurs; whereas at the regions close to Dot1, the propagation between H3K79me2 and H3K79me3 is more likely to occur (Fig 1E, Table 1, and S1C Fig).

3. The “*H4K20me1 –H2A*.*Z*” module is mainly composed of the heterologous “H4K20me1 → H2A.Z” interaction downstream of TSS and the opposite interaction upstream of TSS (S1C Fig). As H2A.Z is known to destabilize nucleosomes[2] and H4K20me1 to condense chromosome[25], the interaction between H4K20me1 and H2A.Z suggests of a balancing effect between the two (Fig 1E, Table 1 and S1C Fig). At TSS downstream, where nucleosomes are well-phased, the “H4K20me1 → H2A.Z” interaction dominates, whereas at TSS upstream, where NFRs mainly reside, the opposite interaction “H2A.Z → H4K20me1” dominates. This suggests that the counteracting effects of H2A.Z and H4K20me1 might be involved in NFRs upstream and nucleosome phasing downstream of TSS.

Overall, the two signal propagation models from TSSs to the upstream and downstream are very similar (S1E Fig), which is consistent with the general symmetry of most TF/histone modification distribution patterns around TSSs. However, in comparison with TSS upstream, the models imply a more complex mechanism of transcription regulation at TSS downstream, as there are some specific interactions downstream (S1F Fig), including “H3K4me3 → H4K20me1”, “H3K79me3 → H2A.Z” and “H3K79me1 → H3K27me3”. Similarly, the two interactions “H3K79me1 → H4K20me1” and “H3K9me3 –H4K20me3” inferred only at TTS upstream (S1F Fig) might be related to region specific regulations.

In addition, the three common modules were obtained again when we performed DBN inference at enhancers based on the directions of information flow assumed as from the center of enhancer regions towards the nearest TSSs (S2A and S2B Fig). It suggests a probable ubiquity of the three modules across active regions in the genome.

### In silico validation of the DBN propagation models

#### Correlation analysis of the three common modules

**Fig 2:**
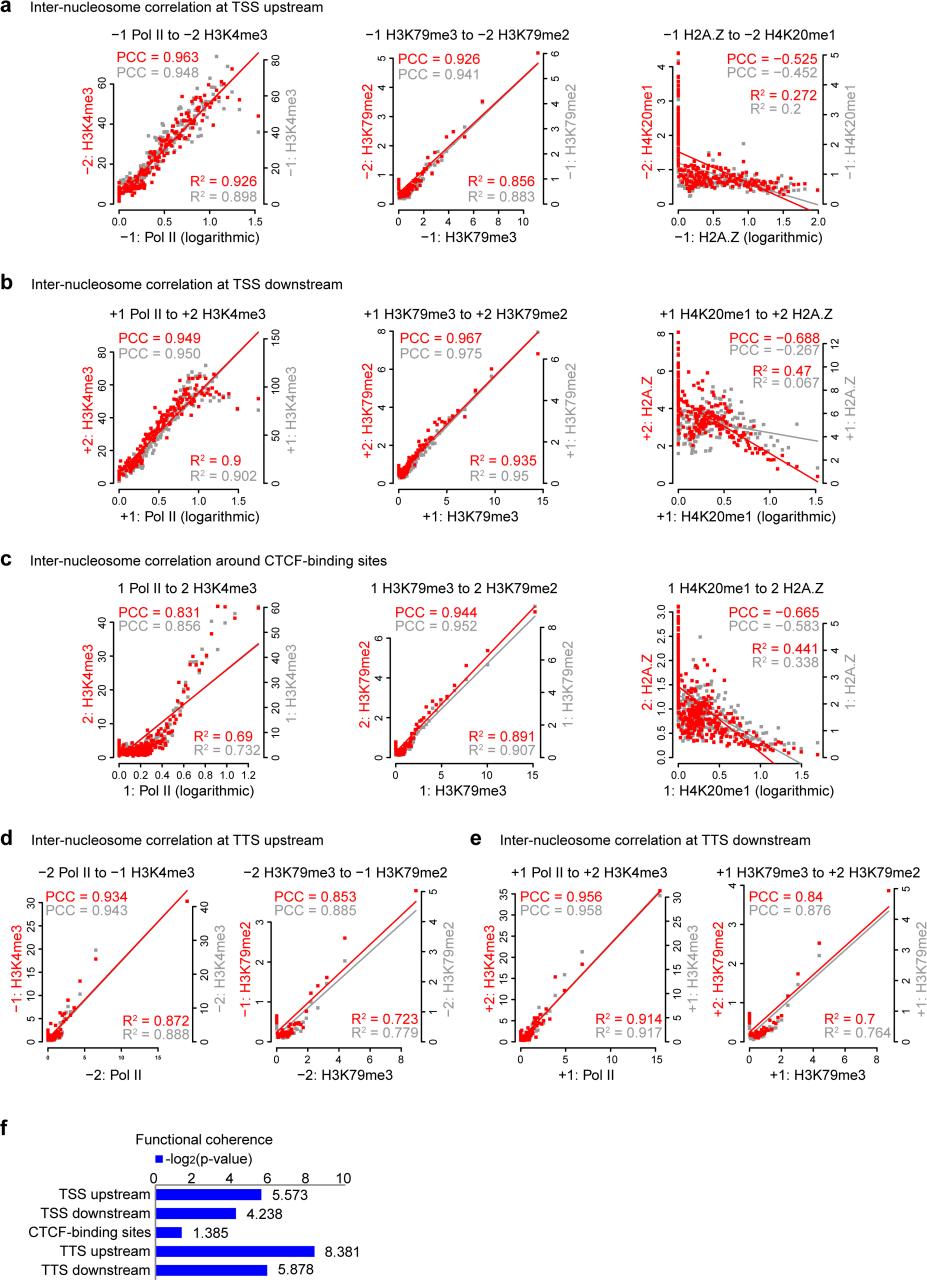
In silico validations of the inter-nucleosomal interactions. (**a-e**) Correlation between/among the factors in each of the three common modules. The correlation between factor A’s level at “before” nucleosomes and factor B’s level at “after” nucleosomes was illustrated by a scatter plot and quantified by Pearson correlation coefficient (PCC, red dots). Each point represents 100 nucleosome pairs grouped as a bin by the factor A levels at “before” nucleosomes. The relationships between “-1” and “-2” nucleosomes in TSS upstream (a), “+1” and “+2” nucleosomes in TSS downstream (b), “1” and “2” nucleosomes to CTCF-binding sites (c), “-2” and “-1” nucleosomes in TTS upstream (d), and between “+1” and “+2” nucleosomes in TTS downstream (e) are shown by scatter plots. Trend lines are fit to linear regression, whose adjusted R^2^ are shown together with PCC. See S3 Fig for more illustration of other nucleosome pairs in TSS, CTCF, and TTS regions. The on-site correlations (between the two different factors’ level at the same “before” nucleosomes) are shown with grey dots for comparison. Some non-linear correlations are illustrated by logarithmic converted Mark-A signals (x-axis) versus raw Mark-B signals (y-axis). (**f**) Co-citation analysis of the functional coherence of the inter-nucleosome consensus networks.

To validate the common heterologous interactions (Fig 1E) inferred by DBN, we analyzed the correlation between/among the different factors (Factor-A signal intensities on a nucleosome versus Factor-B on its neighboring nucleosome) of each module respectively (see Materials and methods for details). A significant positive correlation is revealed between pol II versus H3K4me3, and among different H3K79 methylation stages (red dots in the scatter plots of Fig 2 and S2C Fig, and case-by-case signal profiles in S3 Fig).

More importantly, for the H4K20me1/H2A.Z module, a significant negative correlation for H4K20me1 versus H2A.Z (Fig 2, and more illustration in S3 Fig) is revealed between neighboring nucleosomes. This suggests that although H4K20me1 and H2A.Z generally coexist around active genes at a genome-wide level (S4 Fig), a potential competition between H4K20me1 and H2A.Z at single nucleosome level might balance the nucleosome destabilization by H2A.Z and the chromosome condensation by H4K20me1 in transcription regulation. Notably, the inter-nucleosome anti-correlation between H4K20me1 and H2A.Z at enhancers is much stronger than promoters and CTCF-binding regions (Pearson correlation -0.897 at enhancers versus −0.525, −0.688, and −0.665 at TSS upstream, downstream, and CTCF-binding regions correspondingly, cf. S2C Fig vs. Fig 2A–2C). It suggests a probable ubiquity of the inter-nucleosome anti-correlation between H4K20me1 and H2A.Z across active regions in the genome.

Interestingly, although inter-nucleosome interactions can be also expected to occur on the same nucleosomes, comparing to the correlation between two interactors on the same nucleosomes (see the grey dots in Fig 2), inter-nucleosome correlations between H4K20me1 and H2A.Z are much higher, especially for the TSS +1 to +2 nucleosomes (red versus gray dots in in Fig 2B), suggesting that H4K20me1→ H2A.Z is more specific for inter-nucleosomes along the chromosome than an on-site interaction on the same nucleosome.

#### Co-citation analysis of the biological significance

We used CoCiter (http://www.picb.ac.cn/hanlab/cociter), a co-citation evaluation tool developed by our lab [26], to quantify the significance of biological association between interacting nodes in the nucleosome propagation model. For two terms, CoCiter counts the number of papers that contain both of the two terms in their abstracts, which quantifies the potential correlation level of the two terms. So, by considering commonly used synonyms (Table B in S1 Text), we calculated the pairwise co-citation counts of all the 23 histone modifications/TFs (Table C in S1 Text) (using the CoCiter database on Jan 21, 2013). By simulating 1000 random networks with the same number of “heterologous” edges as background, we quantified the biological significance of the real network by empirical p-value, which is defined as the percentage of randomly assembled networks whose co-citation values are equal to or higher than the real network. The analysis revealed significant literature co-citation (Fig 2F) for the nucleosome propagation networks around TSS/TTS regions (p < 0.05), but not for CTCF regions. The relatively high p-value for the CTCF network might be due to the relatively small number of studies on the CTCF regions.

### Visualizing the H2A.Z and H4K20me1’s gene-wise positive association and nucleosome-wise negative association and their relationship with transcription activity

**Fig 3:**
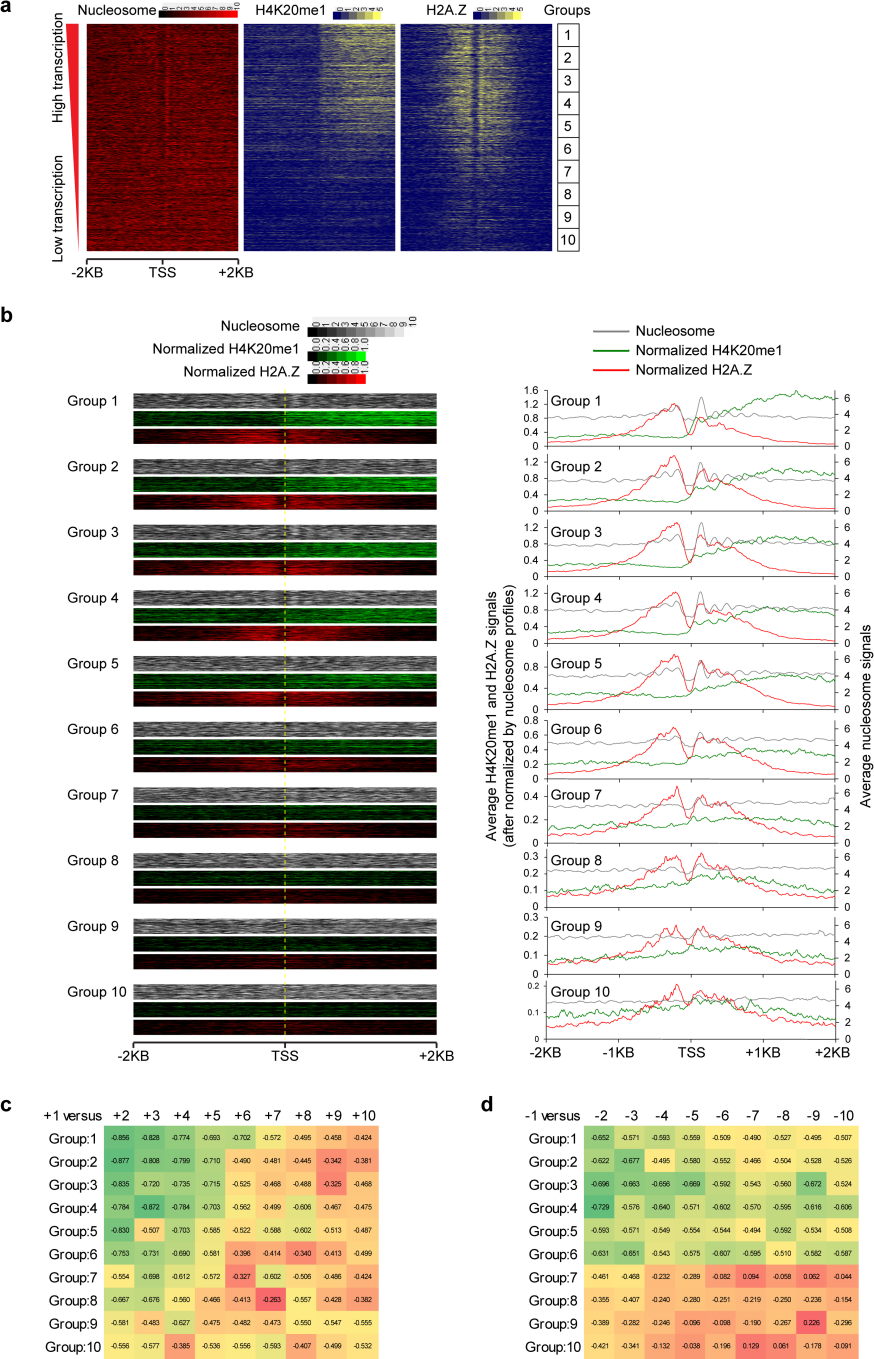
Visualization of H2A.Z and H4K20me1’s gene-wise positive association and nucleosome-wise negative association. (**a**) Nucleosome and H4K20me1/H2A.Z profiles around -2000 ~ +2000 bp of the TSS regions. TSSs are decreasingly ranked by transcription levels, and evenly divided into 10 groups. (**b**) Comparison between normalized H4K20me1 (green) and H2A.Z (red) profiles around TSSs. Raw nucleosome profiles (grey) are shown. (**c**) H4K20me1/H2A.Z correlation for “+1” nucleosome versus surrounding nucleosomes along TSS downstream. For each group, the cross-TSS PCC between “+1” and “+2”, “+3”, …, “+10” nucleosomes were calculated with each bin of 100 TSSs respectively. See S5 Fig for more illustration on different nucleosomes. (**d**) Same as (c), but for H2A.Z/H4K20me1 correlation for “-1” nucleosome versus surrounding nucleosomes along TSS upstream.

To further confirm the authenticity of the global gene-wise positive association and nucleosome-wise negative association between H2A.Z and H4K20me1, we visualized all transcribed genes’ nucleosome, H2A.Z and H4K20me1 intensity profiles at TSS+/- 2 kb sorted by gene expression levels. From the heatmap profiles, we can indeed observe that both H2A.Z and H4K20me1 are higher in highly expressed genes than lowly expressed genes (Fig 3A and 3B). Additionally, for highly expressed genes, after normalizing by the nucleosome signals, H2A.Z is high on the nucleosomes, whereas H4K20me1 is low on the nucleosome but high between nucleosomes (Fig 3B), suggesting an extension of H4K20me1 from the position of the core nucleosome toward internuclesomal region. This is consistent with the finding that H4 tail, which includes the K20 site, interacts with neighboring nucleosomes in multi-nucleosome crystal structures[27, 28]. Consistently, the mutual exclusion is more likely to occur between the nearby nucleosomes rather than between nucleosomes farther apart (Fig 3C and 3D and S5 Fig). Furthermore, the peaks of H2A.Z are higher, while the valleys of H4K20me1 are lower in highly expressed genes compared with lowly expressed genes (Fig 3B).

### The H4K20me1/H2A.Z module is associated with size of nucleosome free region and nucleosome phasing at TSSs

**Fig 4:**
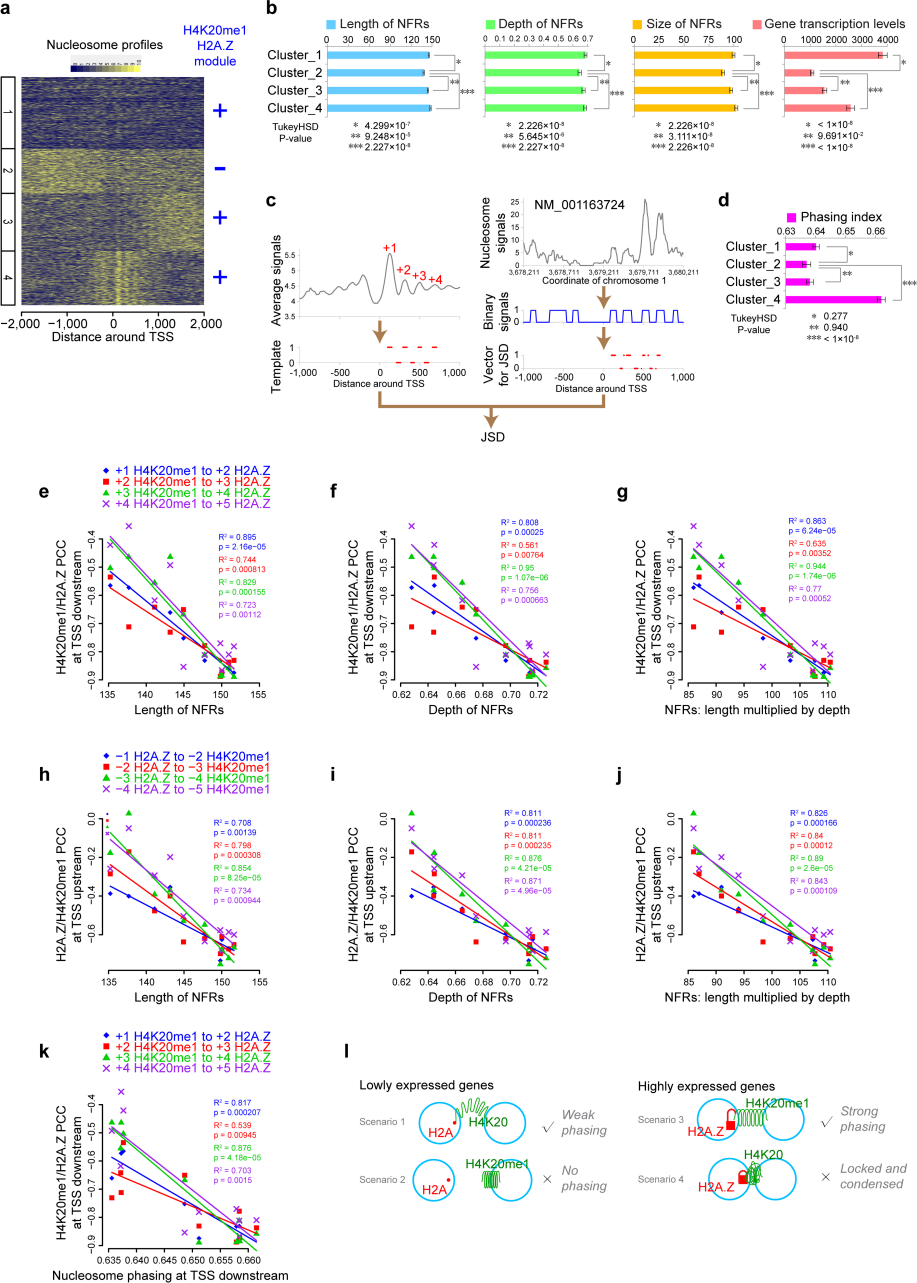
Association of the H4K20me1-H2A.Z interaction with nucleosome profile patterns around TSS. (**a**) Four clusters of the nucleosome profiles in -2000 ~ +2000 bp regions surrounding TSS. The H4K20me1-H2A.Z interaction was obtained in Cluster 1, 3 and 4. (**b**) Length, depth and size of nucleosome free regions, and gene transcription levels for each of the four TSS clusters. The mean and standard error of the mean (SEM) are shown. One-way ANOVA was used for overall comparison, and TukeyHSD test was used to calculate the p-values between Cluster 2 and other clusters. (**c**) Pipeline for calculating Nucleosome Phasing Index. Nucleosome Phasing Index was quantified based on the Jensen-Shannon divergence distance (JSD) between the binary template vector and the binary-converted nucleosome-signal vector of each TSS (see Methods for details). (**d**) Phasing Index for each of the four TSS clusters. Statistical tests were same as (b). (**e-g**) Cross-group linear regression for H4K20me1-H2A.Z anti-correlation versus the NFR length (e), depth (f), and size (length × depth) (g) downstream of TSSs, respectively. Adjusted R^2^ and p-values are labeled on the panels. (**h-j**) Same as (e-g), but for the H2A.Z-H4K20me1 anti-correlation at TSS upstream. (**k**) Cross-group linear regression for H4K20me1-H2A.Z anti-correlation versus nucleosome Phasing Index. (**l**) Mechanistic model of the H2A.Z-H4K20me1 inter-nucleosome interaction. For lowly expressed genes, neither H2A.Z or H4K20me1 is present (Scenario 1), where H2A and H4K20 from neighboring nucleosome can weakly interact to form a weak interaction between nucleosomes and phasing around TSS through the weak acidic patch of H2A; If there exist H4K20me1 (Scenario 2), it would disrupt inter-nucleosome interaction, cause loss of phasing, and expose DNA to spurious transcription, which is an unfavorable situation. For highly expressed genes, the presence of both H2A.Z and H4K20me1 (Scenario 3) will make a flexible and controllable strong interaction between H4K20me1 of the neighboring nucleosome through the strong acidic patch of H2A.Z; Alternatively, if H4K20 is not methylated (Scenario 4), the interaction is too strong to be regulated and has a propensity to form condensed chromatin structure. The strong extended H2A.Z acidic patch is represented by a lock.

H2A.Z is distributed around TSSs, and known to associate with nucleosome free regions (NFRs) at TSSs and nearby sharp nucleosome peaks [13, 29–31]. To test whether the H4K20me1-H2A.Z interaction probably plays a role in forming the special nucleosome profile patterns around TSSs, we classified all TSSs based on the nucleosome profiles within -2 kb to 2 kb of TSSs. By using the BIC-SKmeans algorithm [32], four distinct clusters were identified from these profiles (Fig 4A). After that, the well-phased neighboring nucleosome pairs in each cluster were used for DBN inference, resulting in four similar, stable networks (S6 Fig and Table D in S1 Text). In general, three stable network modules (“*Pol II–H3K4me3*”, “*H4K20me1 –H2A*.*Z*”, and “*H3K79me1*, *2*, *and 3*”) exist in all the four networks, except the lack of the “H4K20me1 → H2A.Z” interaction in the network for Cluster 2, which has weaker patterns of NFRs and nucleosome phasing (Fig 4A and S6A Fig). This suggests that the inter-nucleosomal interaction “H4K20me1 → H2A.Z” is associated with the formation of NFRs and nucleosome phasing around TSS. This is consistent with the significantly shorter “length” (see Materials and methods and Fig 4B; one-way ANOVA P-value = 2.839×10^−11^, and TukeyHSD P-value = 4.299×10^−7^, 9.248×10^−5^, and 2.227×10^−8^ for Cluster 1, 3, and 4 versus Cluster 2 respectively), smaller “depth” (see Materials and methods and Fig 4B; one-way ANOVA P-value = 7.614×10^−15^, and TukeyHSD P-value = 2.226×10^−8^, 5.645×10^−6^, and 2.227×10^−8^ for Cluster 1, 3, and 4 versus Cluster 2 respectively) and smaller “size” (as estimated by *length* × *depth* of NFRs; Fig 4B; one-way ANOVA P-value < 2.2×10^−16^, and TukeyHSD P-value = 2.226×10^−8^, 3.111×10^−8^, and 2.226×10^−8^ for Cluster 1, 3, and 4 versus Cluster 2 respectively) of the NFRs in Cluster 2 compared with the other three clusters. In addition, the disappearance of NFRs corresponds to the decreasing H2A.Z/H4K20me1 signals (see Materials and methods and S6B and S6C Fig). Moreover, the lower gene transcription levels of Cluster 2 compared with the other three clusters (Fig 4B; one-way ANOVA P-value < 2.2×10^−16^, and TukeyHSD P-value < 1×10^−8^, 9.691×10^−2^, and < 1×10^−8^ for Cluster 1, 3, and 4 versus Cluster 2 respectively) are highly consistent with the lack of “H4K20me1 –H2A.Z” interaction in Cluster 2, as H4K20me1 and H2A.Z are activating histone marks [20].

We next quantified nucleosome phasing in these gene clusters using “Phasing Index” for nucleosome profile within TSS ~ TSS +/- 20 kb region for the first four nucleosomes. The Phasing Index is defined the Jensen-Shannon divergence distance (JSD) (Materials and methods) between the binary-converted nucleosome profile (E_1_) downstream of the corresponding TSS (S6D Fig) and a binary template vector (E_0_), representing the “+1” ~ “+4” nucleosome phasing, in which the nucleosome peak region is defined as 1, while valley as 0 (Fig 4C).

Using this Phasing Index, we found that there is low nucleosome phasing in Cluster 2 compared with the other three clusters (Fig 4D), and the level of nucleosome phasing corresponds to the decreasing H2A.Z/H4K20me1 signals (see Materials and methods and S6E and S6F Fig).

We then seek to quantitatively assess the relationship of the anti-correlation between H4K20me1 and H2A.Z at neighboring nucleosomes to NFR size and nucleosome phasing at TSSs. Indeed, not only the H2A.Z or H4K20me1 intensity (S6G–S6J Fig), but also the strength of H4K20me1-H2A.Z anti-correlation is associated with the length, depth, and in particular, size (as estimated by *length* × *depth*) of NFRs (Fig 4E–4J). Similarly, the H4K20me1-H2A.Z anti-correlation at TSS downstream is also significantly associated with the strength of nucleosome phasing (Fig 4K and see also Materials and methods), which is stronger than the association of H4K20me1 intensity with phasing (S6F Fig), but a little weaker than that of H2A.Z (S6F Fig). This suggests a contribution of the H4K20me1-H2A.Z anti-correlation in forming the whole canonical nucleosome pattern, including both on-site/upstream NFRs and downstream nucleosome phasing, which probably plays a vital role in transcription regulation.

### H2A.Z knockdown induced decrease of nucleosome free region and nucleosome phasing at TSSs

**Fig 5:**
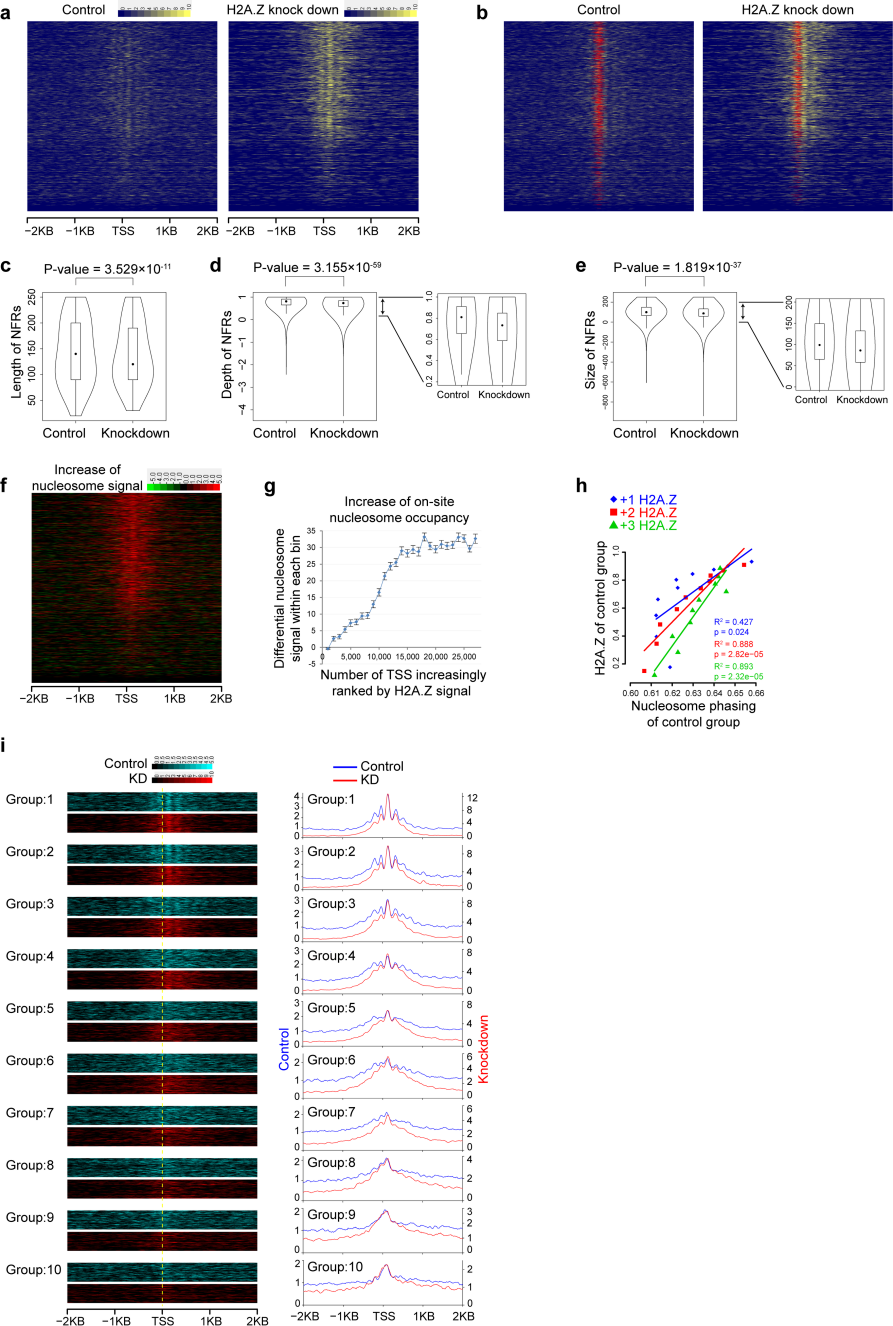
H2A.Z knockdown induced decrease of nucleosome free regions and nucleosome phasing at TSS regions. (**a**) Nucleosome profiles around TSSs in control group and H2A.Z knockdown group. TSSs are decreasingly ranked by their H2A.Z levels (the sum of normalized H2A.Z signal within -2000 to +2000 bp) in control group. (**b**) Nucleosome free regions (NFRs) marked in red color for control and knockdown groups. (**c-e**) Length (c), depth (d), and size (e) of nucleosome free regions. The median and quartile are shown. P-value was calculated by one-tailed t-test. (**f**) H2A.Z knockdown induced increase of nucleosome signals around TSS versus control group. The differential nucleosome profiles between H2A.Z knockdown and control group were calculated by DANPOS with the setting of “quantile normalization”. (**g**) Increase of “on-site” nucleosome occupancy on TSSs. TSSs are increasingly ranked by their H2A.Z levels in control groups, and the increase of “on-site” nucleosomes is plotted to show the mean ± SEM values within each bin of 1000 TSSs. Spearman correlation was calculated between the difference of “on-site” nucleosome occupancy at TSSs (the sum of differential nucleosome signals within -90 to +20 bp around TSS) versus the H2A.Z level (the sum of H2A.Z normalized-signals within -2000 to +2000 bp around TSS) of the corresponding TSSs in control group. (**h**) Cross-group linear regression for H2A.Z levels versus the nucleosome Phasing Index of control sample. Adjusted R^2^ and p-values are labeled on the panels. (**I**) Comparison of nucleosome profiles between control and knockdown samples. Nucleosome profiles are aligned within -2000 to +2000 bp around TSSs in a 10 bp resolution. TSSs (>20000 TSSs having non-zero H2A.Z signals at “+1” nucleosomes) are decreasingly ranked by H2A.Z levels at “+1” nucleosomes, and evenly divided into 10 groups.

As H2A.Z is distributed around TSSs, and known to associate with nucleosome free regions (NFRs) at TSSs and nearby sharp nucleosome peaks [13, 29–31], to confirm the critical role of H2A.Z in the maintenance of NFRs at TSSs, we examined the MNase-seq dataset for H2A.Z knockdown (KD) in mouse embryonic stem cells (mESC) [33]. The nucleosome signal intensity around TSSs was indeed increased by H2A.Z KD (Fig 5A and 5F), accompanied by a decrease in the length, depth, and size of NFRs around TSS (Fig 5B–5E). Furthermore, the significant cross-TSS correlation (Spearman correlation coefficient = 0.426, P-value < 2.2×10^−16^) between the H2A.Z levels and the KD induced “on-site” nucleosome-signal increase on TSSs (Fig 5F and 5G) is consistent with the established role of H2A.Z in attenuating nucleosome occupancy at TSSs [13, 29–31]. In addition, the H2A.Z levels are significantly associated with the strength of nucleosome phasing of control sample (Fig 5H). Even more, the KD of H2A.Z induced a decrease of nucleosome phasing at TSS regions (Fig 5I and S7A and S7B Fig). Therefore, KD of H2A.Z, i.e. a perturbation of H2A.Z-H4K20me1 interaction, resulted in the decrease of NFRs and nucleosome phasing at TSS regions, supporting the role of H2A.Z-H4K20me1 module in forming and maintenance of canonical nucleosome patterns at TSS regions. Finally, the H2A.Z KD also induced a decrease of nucleosome phasing around CTCF-binding sites (S7C and S7D Fig), implying the generality of the role of H2A.Z-H4K20me1 interaction in forming/maintaining nucleosome phasing.

## Discussions

In this study, we developed a DBN learning algorithm to infer heterologous inter-nucleosomal interactions or communications among 23 histone modifications, variants, and TF-binding at single nucleosome level.

Supporting the inferred internucleosomal “Pol II → H3K4me3” interaction, Zhang *et al*. have proposed a dynamic interaction mechanism between Pol II and H3K4me3 in budding yeast: The transcription inhibition is triggered by certain ‘transcription stress’, which selectively evicts nucleosomes with H3K4me3 modification, or displaces them toward 3’ position of the gene [34]. Therefore, the internucleosomal “Pol II → H3K4me3” interaction implicates that Pol II can trigger the H3K4me3 at neighboring nucleosome, which then enhances the disassembly of nucleosomes to facilitate the sliding of Pol II along the chromatin for transcription process.

Another stable pattern of interaction in our consensus networks is the heterologous regulations among the three states of H3K79 methylation. The mono-, di-, and trimethylation of H3K79 are all catalyzed by Dot1L [24, 35, 36]. Specifically, Dot1L is preferentially accumulated at TSSs of active genes, which is correlated with the abundance of H3K79me2 and H3K79me3 at this region. The distribution of H3K79me3 has a sharp peak at the slight downstream position of TSSs, which is similar to Dot1L. Yet, the sharpness of H3K79me2/1 decreases, the shape of the distribution is similar to H3K79me3. Consistent with the concentration effect of Dot1L, a nonprocessive methylation mechanism is proposed by Frederiks F, *et al*. [24] (see Fig 2 therein for a more detailed description), which might explain the propagation pattern of the three states of H3K79 methylation.

We also inferred a stable H4K20me1 → H2A.Z interaction at nearby nucleosomes around TSS regions. Several studies [2, 29–31, 37–40] have reported mechanisms of H2A.Z in the regulation of transcription events around TSS in several different species. Although the detailed molecular mechanism may vary in different species, it is clear that H2A.Z occupancy is preferentially located at the nucleosomes flanking the “nucleosome free regions” (NFR) at TSS. On the other hand, H4K20me1 regulates chromosome condensation [25].

Our finding of the anti-correlation between H4K20me1 and H2A.Z on neighboring nucleosomes highlights a new pattern of epigenetic regulation, and sheds new light on the long-standing puzzle why and how the chromatin repressive mark H4K20me1 is enriched at the TSS regions of active genes. Despite H4K20me1 is overall enriched together with some other active histone marks at the TSS of active genes, such as H2A.Z, at single nucleosome levels, H4K20me1 is mutually exclusive with neighboring H2A.Z. We speculate that the general coexistence of H4K20me1 and H2A.Z at long range around the actively transcribed genomic regions might balance the nucleosome eviction with a nucleosome re-phasing regulation, where at single nucleosome level, the occupancy or eviction of nucleosome probably depends on the competition between H4K20me1 and H2A.Z thus resulting in a dynamic balance and phasing in the local condensation-decondensation of chromatin. This hypothesis is consistent with the crystal structure of regular and H2A.Z containing nucleosomes, where the acidic patch on both H2A and H2A.Z interacts with the highly positively charged H4 tail on the neighboring nucleosome[27, 28]. The acidic patch is much more extended in H2A.Z, making it potentially interact with the H4 tail more strongly to compact the chromosome. Acetylation of the H4 tail can disrupt nucleosome compaction, probably through the disruption of H4 tail binding to neighboring nucleosomes[41]. Similarly, we speculate that methylation of K20 on the H4 tail may reduce its positive charge, avoiding a locked binding or allowing more flexible and controllable binding of H2A.Z to the neighboring nucleosome, which is essential for both eviction and strong phasing of the nucleosome to form highly ordered open chromatin structure (Fig 4I). This model can well explain the high positive correlation of H2A.Z and H4K20me1 across TSSs and their association with high transcription activity. The anti-correlation between H2A.Z and H4K20me1 on neighboring nucleosomes is equally intriguing, which might be attributed to an enzymatic activity associated with H2A.Z rendering the neighboring H4K20me1 to other modification states.

Consistent with the DBN inferred, the intensity of interacting histone modifications and variations at the single neighboring nucleosomes show significant correlations (Fig 2 and S2C Fig and S3 Fig). Yet, more powerful than canonical correlation analyses, the DBN identifies not only linear correlation (e.g. among H3K79me1/2/3, see the scatter plotting in Fig 2) but also non-linear correlation (e.g., Pol II-H3K4me3 and H2A.Z-H4K20me1 interactions, see the scatter plotting in Fig 2), which could not be readily detected by linear correlations.

### Conclusions

The consensus networks inferred by the DBN algorithm at single nucleosome level uncovered robust and stable inter-nucleosome propagations and their modular structures. Among the novel interactions, the H2A.Z-H4K20me1 anti-correlation uncovered a new potential mechanism in forming and maintenance of nucleosome phasing and in balancing the space/distance between neighboring nucleosomes. Our new method for *ab initio* inference of inter-nucleosome propagating signals at single nucleosome level will be readily applicable to delineate epigenetic signaling mechanisms around many other functional genomic elements and will help to decipher the mechanisms of dynamic chromatin remodeling events.

## Materials and methods

### Data sets

Tag coordinate bed files for MNase-digestion sequencing data of human CD4+ T cells [13] was downloaded from National Heart Lung and Blood Institute (NHLBI), National Institutes of Health (NIH) (http://dir.nhlbi.nih.gov/papers/lmi/epigenomes/hgtcellnucleosomes.aspx). Tag coordinate bed files for MNase-digestion ChIP-seq data [20] was downloaded from another webpage in NHLBI, NIH (http://dir.nhlbi.nih.gov/papers/lmi/epigenomes/hgtcell.aspx) for the distribution of 23 types of histone modifications and TF-binding: *H3K4me1*, *H3K4me2*, *H3K4me3*, *H3K9me1*, *H3K9me2*, *H3K9me3*, *H3K27me1*, *H3K27me2*, *H3K27me3*, *H3K36me1*, *H3K36me3*, *H3K79me1*, *H3K79me2*, *H3K79me3*, *H3R2me1*, *H3R2me2*, *H4K20me1*, *H4K20me3*, *H4R3me2*, *H2BK5me1*, *H2A*.*Z*, *Pol II*, *and CTCF*. Gene expression microarray data for human CD4+ T cells [13] was downloaded from the GEO repository with accession number GSE10437. The coordinate information of human TSS/TTS was downloaded from UCSC repository webpage (http://hgdownload.cse.ucsc.edu/goldenPath/hg18/database/refFlat.txt.gz) on July 30, 2012. The coordinate information of human CTCF-binding sites [42] was downloaded from (http://bioinformatics-renlab.ucsd.edu/rentrac/wiki/CTCF_Project), and transferred into hg18 system. The ChromHMM predicted coordinate information of human enhancers in CD4 T memory primary cells were obtained from the RoadMap repository. Tag coordinate bed files for MNase-digestion sequencing data of the H2A.Z KD and control group for mES cells [33] was downloaded from GEO repository with accession number GSM849959 (ftp://ftp.ncbi.nlm.nih.gov/geo/samples/GSM849nnn/GSM849959/suppl/GSM849959_GA2807_CMT1_shH2A.Z-2d_MNase_0.1U_r520l2.bed.gz) and GSM849958 (ftp://ftp.ncbi.nlm.nih.gov/geo/samples/GSM849nnn/GSM849958/suppl/GSM849958_GA2804-CMT1-shLuc-a-MNase-0.1U_r520l1.bed.gz) respectively. Tag coordinate bed files for H2A.Z ChIP-seq data of the wild type mES cells [33] was downloaded from GEO repository with accession number GSM849928 (ftp://ftp.ncbi.nlm.nih.gov/geo/samples/GSM849nnn/GSM849928/suppl/GSM849928_GA1141-mouse-ES-H2A.Z-Final-DNA-m1-r338l6r356l7_noDup-pool.bed.gz). The coordinate information of mouse TSSs was downloaded from UCSC repository webpage (http://hgdownload.soe.ucsc.edu/goldenPath/mm8/database/refFlat.txt.gz) on Dec 17, 2014. DANPOS [43], version 2.1.2, was downloaded from http://code.google.com/p/danpos/ on May 14, 2013.

### Nucleosome selection and preparation for dynamic Bayesian network inference

Genome-wide nucleosome positions were detected from MNase-digestion sequencing data [13] by the iNPS software [23]. Regularly phased nucleosomes were selected as preliminary candidates for DBN training according to two of the following three criterions. **(1)** A nucleosome has a ‘width’ (the length between two consecutive inflection points in the scoring profile) between 70 ~ 90 bp. **(2)** This nucleosome also has ‘adjacency distances’ (the distance from the center point of this nucleosome to the center point of its left/right neighboring nucleosome) between 160 ~ 400 bp. **(3)** The type of this nucleosome is “MainPeak” based on the identification of iNPS according to the shapes of detected nucleosome peaks. Then, from these nucleosome candidates, each pair of neighboring nucleosomes phased around TSS or TTS or CTCF-binding sites was collected for DBN learning. Note that some nucleosome pairs (< 20%), belonging to two or more TSS/TTS/CTCF regions, were excluded to avoid potential signal spillover.

Additionally, we performed DBN inference at three “center-inclusion levels” respectively by including or excluding the nucleosome pairs overlapping with the center of TSS/TTS/CTCF regions as shown in S1A and S1B Fig.

### Learning dynamic Bayesian networks

To study the information propagation principles between neighboring nucleosomes, we need to learn the structure of the transition network in the DBN model. As shown in (Friedman et al. 1998), for stationary time processes, the transition network can be conveniently represented by a template network which is repeated at consecutive time frames (*t*−1,*t*) with 2*n* nodes (*X*[*t*−1],*X*[*t*]), where *X* = {*X*_1_,*X*_2_,⋯,*X*_*n*_} represents the set of nodes/variables being considered (the level of histone modifications/TF-bindings at each nucleosome), *t* represents the ‘time’ index (the nucleosome order along an upstream or downstream direction in a TSS/TTS/CTCF region). To learn the DBN model, we first curate a training set *D* consisting of all the ordered nucleosome sequences for the TSS/TTS/CTCF genomic regions. Each sequence is denoted by *X*^*l*^, where *l* is the index of the sequence. Then, we generate an alternative set of training data *D*' by concatenating values of *X* at two consecutive time frames in all the ordered sequences, i.e., each data item in this new set is 2*n* dimensional and has the form (*X*^*l*^[*t*−1],*X*^*l*^[*t*]), where all valid indices (*l*,*t*) in *D* have been included. An important result we conclude from (Friedman et al. 1998) is that the scoring function of a transition network given *D* is equivalent to the scoring function for the corresponding constrained BN given *D*' (plus a constant), where edges *among the first n nodes X*[*t*−1] and *edges from the second n nodes X*[*t*] *to the first n nodes X*[*t*−1] are prohibited in the constrained BN (i.e., only edges within *X*[*t*] or from *X*[*t*−1] to *X*[*t*] are allowed).

In our DBN learning task, all the variables are real-valued. This is different from (Friedman et al. 1998), which only considered DBN learning on discrete data sets. Thus, instead of using traditional BN scoring functions for discrete data, we use the Kernel-based BN learning approach implemented in the SeqSpider software (Liu et al., Cell Res 2013), which well supports modeling the interactions between real-valued variables. Note that the *a priori* structural constraints described above must be specified before executing the program. Moreover, all the default parameter settings of SeqSpider are used except *λ* is set to 2.0 (which weights the penalty term in the kernel-based scoring function) to make the number of output edges at a reasonable range. Finally, as a common practice in BN learning, we need to parse edges in the constrained Bayesian network into compelled (directed) / non-compelled (undirected) edges (which collectively form a partially directed acyclic graph, a.k.a., PDAG) to distinguish identifiable causal relationships and non-identifiable ones. This is done by taking into account the constraints mentioned above and the BN structure itself using Meek’s rule (Meek 1995). After this step, only compelled edges represent potential causal relationships consistent with training data and the constraints, since only the directions of these edges are invariant within all equivalent BN structures.

The DBN inference is run on 10 random sub training-sets, each containing 90% of input data. Then, we obtain 10 PDAG networks accordingly. As a default operation in SeqSpider, edges that appear in ≥ 7 PDAGs are selected to constitute a cross validated network. The stability for obtaining this cross validated network is measured by a “Receiver Operator Characteristic (ROC) curve”, also defined in (Liu et al., Cell Res 2013). The area under the ROC curve (AUC) is used to quantify stability of the network.

### Correlation analysis for inferred interactions

Within -2000 to +2000 bp around each TSS/TTS, the nucleosomes at upstream and downstream regions were sequentially indexed with “-m, -m+1, -m+2, …, -3, -2, -1” and “1, 2, 3, …, n-2, n-1, n” respectively. While within -/+2000 bp around each CTCF-binding sites, the nucleosomes at two flanking sides were symmetrically indexed with “n, n-1, n-2, …, 3, 2, 1” and “1, 2, 3, …, n-2, n-1, n”, respectively, as these regions have no definite directionality.

Then, for each stable interaction from Mark-A to Mark-B, correlation was evaluated between the Mark-A and Mark-B signals at “before” and “after” nucleosomes across the mean values in each bin of 100 nucleosome pairs decreasingly ranked by the Mark-A signal intensities at “before” nucleosomes. Only when either Mark-A signal value of “at-before” nucleosome or Mark-B signal value of “at-after” nucleosome is not zero, the nucleosome pair is considered. Note that a non-linear correlation between Mark-A and Mark-B was evaluated between logarithmic converted signals of Mark-A and raw signals of Mark-B.

### Length and depth of nucleosome free region

For each TSS region, the nucleosome free region (NFR) was represented by the region between the nearby upstream (“-1”) and downstream (“+1”) nucleosome peaks flanking the TSS. Based on the definition, the length of NFR for each corresponding TSS was quantified respectively. And the depth of NFR was quantified by using the following formula, in which the denominator is the average signal of the corresponding regions (with 10 bp resolution).

$$1-\frac{nucleosome\_free\_region\_signal}{(nearby\_upstream\_peak\_signal+nearby\_downstream\_peak\_signal)\times 0.5}$$

Note that a TSS was not used for the analysis of NFRs, if the TSS did not have either “-1” or “+1” nucleosome, or if the width between them was more than 250 bp.

### Nucleosome Phasing Index at TSS downstream

The average nucleosome profile around >20,000 TSSs were calculated. Then, a binary vector (in a 10 bp resolution) was used to represent the “+1” ~ “+4” nucleosome phasing template, in which the region corresponding to the peak (+100 ~ +159 bp, +290 ~ +349 bp, +480 ~ +529 bp, and +660 ~ +719 bp) on the average profile was scored as 1, while valley as 0 (+210 ~ +269 bp, +390 ~ +439 bp, and +560 ~ +619 bp), leaving a gap of 20 ~ 50 bp between a peak and its nearby valley.

On the other hand, the raw nucleosome profiles for the genomic regions corresponding to the template vector were converted to binary signals (in a 10 bp resolution) by scoring the peak and valley (based on iNPS detection) regions as 1 and 0, respectively.

Then, for each TSS, the Phasing Index was defined as the Jensen-Shannon divergence distance (JSD) between the binary-converted nucleosome profile (E_1_) downstream of the corresponding TSS and the binary template vector (E_0_):

$$JSD(E_{0},E_{1})=1-\sqrt{H(\frac{P_{0}+P_{1}}{2})-\frac{H(P_{0})+H(P_{1})}{2}}$$

Where P_0_ and P_1_ are the two discrete probability distributions normalized from the profile vectors E_0_ and E_1_, where *H* is the entropy of a discrete probability distribution, and *n* represents the total number of 10-bp bins in the template: $H(P)=-\sum_{i=1}^{n}p_{i}\log(p_{i})$, with *P* = {*p*_1_,*p*_2_,⋯,*p*_*n*_}, 0 ≤ *p*_*i*_ ≤1, $\sum_{i=1}^{n}p_{i}=1$.

### Association of H4K20me1-H2A.Z anti-correlation with TSS nucleosome patterns

The TSSs were decreasingly sorted by their transcription levels, and evenly divided into 10 groups. For each group, Pearson correlation coefficient was calculated between H4K20me1 and H2A.Z signals at corresponding “before” and “after” (“+1” and “+2”, “+2” and “+3”, “+3” and “+4”, and “+4” and “+5”) nucleosomes across the mean values in each bin of 100 nucleosome pairs decreasingly ranked by the intensities of the histone mark at “before” nucleosome. Here, only the nucleosome pairs located within ±2000 bp around TSS were used. Then, H4K20me1-H2A.Z correlations (the Pearson correlation coefficient values between logarithmic Mark-A and raw Mark-B (see the section “Correlation analysis for inferred interactions” of Methods) within each of the 10 groups) was linearly regressed to the length, depth or size of NFR, or Phasing Index. The adjusted R^2^ and P-values were used for significance evaluation.

## Supporting information

S1 Fig Inter-nucleosome propagation. **(a)** Three “center-inclusion levels” for the selection of neighboring nucleosome pairs for DBN inferring at TSSs or CTCF-binding regions. Center-inclusion level 1 only includes the neighboring nucleosome pairs completely located at either side of TSS/CTCF center. Center-inclusion level 2 includes all the pairs of level 1, and together with pairs that have one nucleosome overlapping with the TSS/CTCF center. Center-inclusion level 3 includes all the pairs of level 2, together with the nucleosome on the other side of the TSS/CTCF center, i.e. it includes all the neighboring nucleosome pairs around TSS/CTCF region. (**b**) Three “center-inclusion levels” for the selection of neighboring nucleosome pairs for DBN inferring at TTSs. Unlike in panel (a), directions at both sides of TTS follow the direction of transcription. (**c**) Consensus networks of inter-nucleosome propagation at TSS upstream, TSS downstream, TTS upstream, TTS downstream and around CTCF-binding sites, respectively. Pink nodes indicate the histone modifications or TF-binding at “before” nucleosomes, and green nodes indicate those at neighboring “after” nucleosomes. DBN parameter “reg” was set to 2. (**d**) Stability validation of networks by Receiver Operator Characteristic (ROC) curve. Stability validation of networks in panel (c) respectively. The AUC (>0.96) of every DBN network indicates that all the DBN networks used for consensus network building are very stable. “CIL” is the short form of “center-inclusion levels”. (**e**) Similarity among consensus networks of TSS, TTS and CTCF regions: overlap between the networks for TSS upstream and downstream; overlap between the networks for TTS upstream and downstream; overlap among the network of TSS, TTS and CTCF regions. **(f)** Modules specific for TSS downstream and TTS upstream network respectively.
(TIF)

S2 Fig Inter-nucleosome propagation at enhancers. (**a**) Schematic diagrams of the direction of the modeled signal propagation at enhancer. (**b**) Consensus networks of inter-nucleosome propagation at enhancers. (**c**) Correlation between/among the factors in each of the three common modules. The analysis was the same as Fig 2, but between “+1” and “+2” nucleosomes at enhancer regions by the direction from the center of enhancer regions to the nearest TSSs. The correlation between factor A’s level at “before” nucleosomes and factor B’s level at “after” nucleosomes was illustrated by a scatter plot and quantified by Pearson correlation coefficient (PCC, red dots), while the on-site correlations (between the two different factors’ level at the same “before” nucleosomes) are shown with grey dots for comparison.
(TIF)

S3 Fig Heatmaps visualizing the inter-nucleosome correlation. (**a**) Inter-nucleosome correlation for the propagation “Pol II → H3K4me3” from “-1” to “-2” nucleosomes at TSS regions by the direction from TSS center to upstream. The profiles of nucleosome, Pol II, and H3K4me3 signals were mapped to the -1000 to +1000 bp windows around each nucleosome pairs with a 10 bp resolution. The lines (or the corresponding TSSs) were ranked by Pol II signal of the “-1” nucleosome. (**b-c**) Same as (a), but for “H3K79me3 → H3K79me2” and “H2A.Z → H4K20me1” respectively. (**d-f**) Same as (a-c), but for the propagations from “+1” to “+2” nucleosomes at TSS regions by the direction from TSS center to downstream. (**g-i**) Same as (a-c), but from “1” to “2” nucleosomes around CTCF-binding regions in the direction from CTCF-binding sites to two flanking side. (**f and i**) Same as (c), but for the propagation “H4K20me1 → H2A.Z”. (**j-k**) Same as (a-b), but for the propagation from “-2” to “-1” nucleosomes at TTS regions by the direction from TTS upstream to TTS center. (**l-m**) Same as (a-b), but for the propagation from “+1” to “+2” nucleosomes at TTS regions by the direction from TTS center to TTS downstream.
(TIF)

S4 Fig Overall H4K20me1 and H2A.Z signals around TSS regions. (**a**) H4K20me1 and H2A.Z signal profiles around TSSs. TSSs are decreasingly ranked by transcription levels. The profiles are in a 10 bp resolution within -2000 to +2000 bp around TSSs. (**b**) Cross-TSS correlation between H4K20me1 and H2A.Z. TSSs are increasingly ranked by transcription levels. Each point in the scatter plotting represents 100 TSSs grouped as a bin. The total H4K20me1 and H2A.Z signal intensities (after normalized by nucleosome signals) are quantified within -2000 to +2000 bp around TSS, and the mean value of each bin of 100 TSSs are shown. The cross-TSS Pearson correlation coefficient (PCC) is 0.811. (**c-d**) Similar as (a-b), but for the H4K20me1 and H2A.Z signals within -1000 to +1000 bp around the “+1/+2” nucleosome pairs of TSS downstream regions. The lines of heatmaps (c) are decreasingly ranked by the overall normalized H4K20me1 signals around each “+1/+2” nucleosome pair. PCC is 0.794 in (d). (**e-f**) Similar as (a-b), but for the H2A.Z and H4K20me1 signals within -1000 to +1000 bp around the “-1/-2” nucleosome pairs of TSS upstream regions. The lines of heatmaps (e) are decreasingly ranked by the overall normalized H2A.Z signals around each “-1/-2” nucleosome pair. PCC is 0.822 in (f).
(TIF)

S5 Fig Comparison of the H4K20me1/H2A.Z correlation at nucleosome pairs with different distances around TSSs with different transcription levels. (**a**) H4K20me1/H2A.Z correlation at “+2” nucleosome versus surrounding nucleosomes along TSS downstream. Group 1~10 were obtained by decreasingly ranking TSSs according to transcription levels and evenly dividing them into 10 groups, as illustrated in Fig 3A. For each group, the cross-TSS Pearson correlation coefficient between “+2” and “+3”, “+4”, …, “+11” nucleosomes were calculated with each bin of 100 TSSs respectively. (**b-c**) Same as (a), but for “+3” (b) and “+4” (c) nucleosomes respectively. (**d-f**) Same as (a-c), but for H2A.Z/H4K20me1 correlation at “-2”, “-3” and “-4” nucleosome versus surrounding nucleosome along TSS upstream.
(TIF)

S6 Fig DBN networks for different patterns of nucleosome profiles around TSS regions, and the association of H4K20me1/H2A.Z with nucleosome patterns around TSS regions. (**a**) The inter-nucleosome propagation network for each of the four clusters in Fig 4A. The DBN parameter “reg” was set to 2. The “H4K20me1 → H2A.Z” module is marked with box. (**b**) The H4K20me1/H2A.Z profiles within -2000 ~ +2000 bp regions surrounding TSS for the four nucleosome profile clusters shown in Fig 4A. (**c**) The total H2A.Z and H4K20me1 signal intensity (after normalized by nucleosome signals) within -2000 ~ +2000 bp regions surrounding TSS in each of the four clusters. The mean and standard error of the mean (SEM) are shown. One-way ANOVA was used for overall comparison, and TukeyHSD test was used to calculate the p-values between Cluster 2 and other clusters. (**d**) Binary nucleosome signals for the four TSS clusters. (**e**) Binary nucleosome signals for the TSSs decreasingly ranked by transcription levels. The TSSs are evenly divided into 10 groups. (**f**) Cross-group linear regression for H4K20me1 and H2A.Z levels (after normalized by nucleosome profiles) versus the strength of nucleosome phasing respectively. (**g**) Cross-group linear regression for H4K20me1 levels (after normalized by nucleosome profiles) at TSS downstream versus the length, depth, and size (length-multiplied-by-depth) of nucleosome free regions, respectively. Adjusted R^2^ and P-values are labeled on the panels. (**h**) Same as (g), but for H2A.Z at TSS downstream. (**i-j**) Same as (g-h), but for the H4K20me1 (i) and H2A.Z (j) intensities at TSS upstream.
(TIF)

S7 Fig H2A.Z knockdown induced decrease of nucleosome phasing. (**a**) Comparison of nucleosome profiles around TSSs between control and KD samples. Nucleosome profiles are aligned within -2000 to +2000 bp around TSSs in a 10 bp resolution. TSSs (having non-zero H2A.Z signals at “+2” nucleosomes) are decreasingly ranked by their H2A.Z levels at “+2” nucleosomes, and evenly divided into 10 groups. (**b**) Same as a, but based on the H2A.Z signals at “+3” nucleosomes. (**c-d**) Similar as a-b, but for the nucleosome profiles around CTCF-binding sites. (**c**) CTCF-binding sites (having non-zero H2A.Z signals at “1” nucleosomes) are decreasingly ranked by their H2A.Z levels at “1” nucleosomes, and evenly divided into 10 groups. (**d**) Same as c, but based on the H2A.Z signals at “2” nucleosomes.
(TIF)

S1 Text Supplementary tables. (DOCX)
