# Supplementary figures and images for: Inter-nucleosomal communication between histone modifications for nucleosome phasing
Source: PLoS Comput Biol. 2018 Sep 6;14(9):e1006416. doi: 10.1371/journal.pcbi.1006416 (PMC6126837; doi:10.1371/journal.pcbi.1006416)

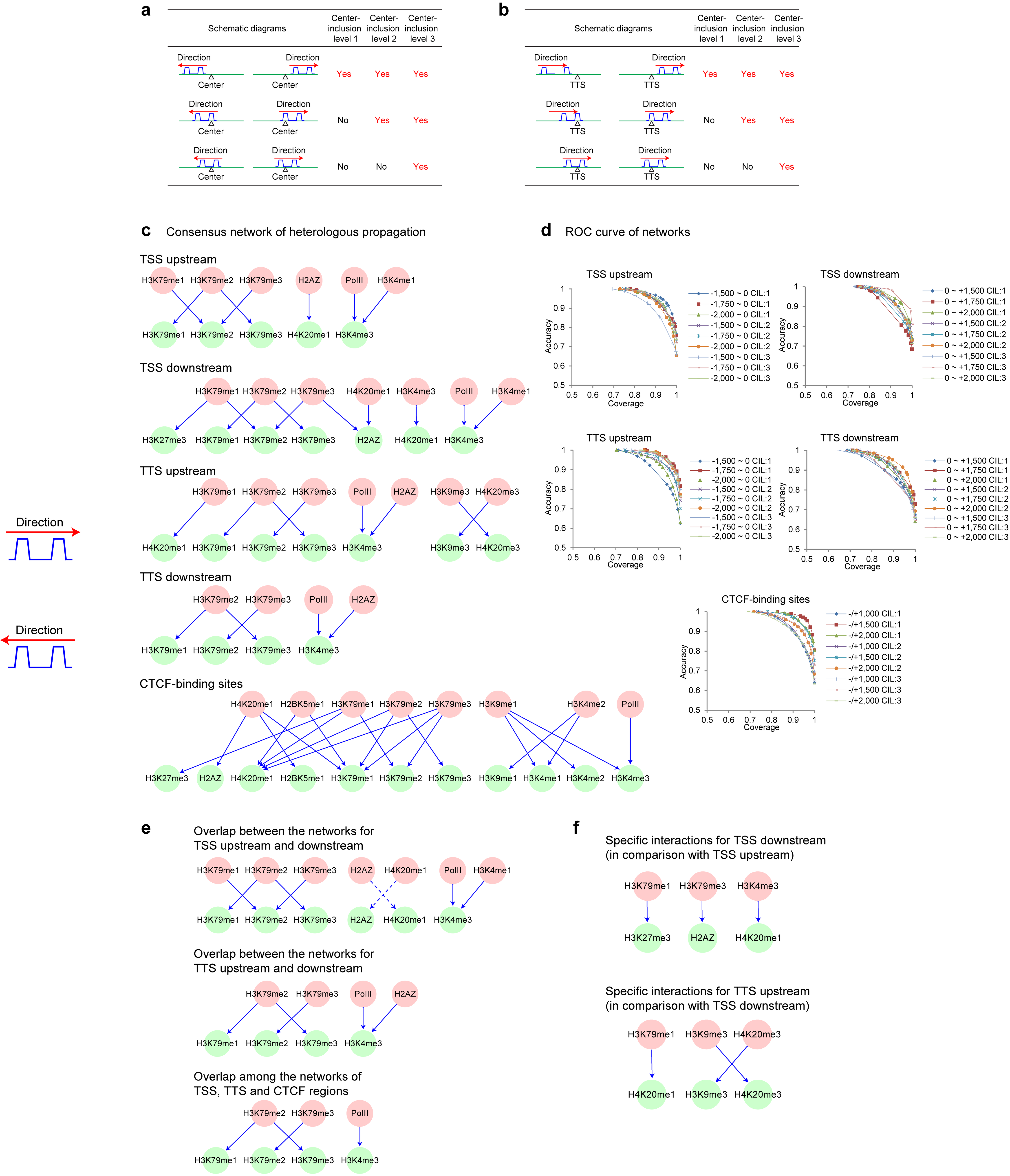

Supplement: S1 Fig — (a) Three “center-inclusion levels” for the selection of neighboring nucleosome pairs for DBN inferring at TSSs or CTCF-binding regions. Center-inclusion level 1 only includes the neighboring nucleosome pairs completely located at either side of TSS/CTCF center. Center-inclusion level 2 includes all the pairs of level 1, and together with pairs that have one nucleosome overlapping with the TSS/CTCF center. Center-inclusion level 3 includes all the pairs of level 2, together with the nucleosome on the other side of the TSS/CTCF center, i.e. it includes all the neighboring nucleosome pairs around TSS/CTCF region. (b) Three “center-inclusion levels” for the selection of neighboring nucleosome pairs for DBN inferring at TTSs. Unlike in panel (a), directions at both sides of TTS follow the direction of transcription. (c) Consensus networks of inter-nucleosome propagation at TSS upstream, TSS downstream, TTS upstream, TTS downstream and around CTCF-binding sites, respectively. Pink nodes indicate the histone modifications or TF-binding at “before” nucleosomes, and green nodes indicate those at neighboring “after” nucleosomes. DBN parameter “reg” was set to 2. (d) Stability validation of networks by Receiver Operator Characteristic (ROC) curve. Stability validation of networks in panel (c) respectively. The AUC (>0.96) of every DBN network indicates that all the DBN networks used for consensus network building are very stable. “CIL” is the short form of “center-inclusion levels”. (e) Similarity among consensus networks of TSS, TTS and CTCF regions: overlap between the networks for TSS upstream and downstream; overlap between the networks for TTS upstream and downstream; overlap among the network of TSS, TTS and CTCF regions. (f) Modules specific for TSS downstream and TTS upstream network respectively. (TIF) [file pcbi.1006416.s001.tif]

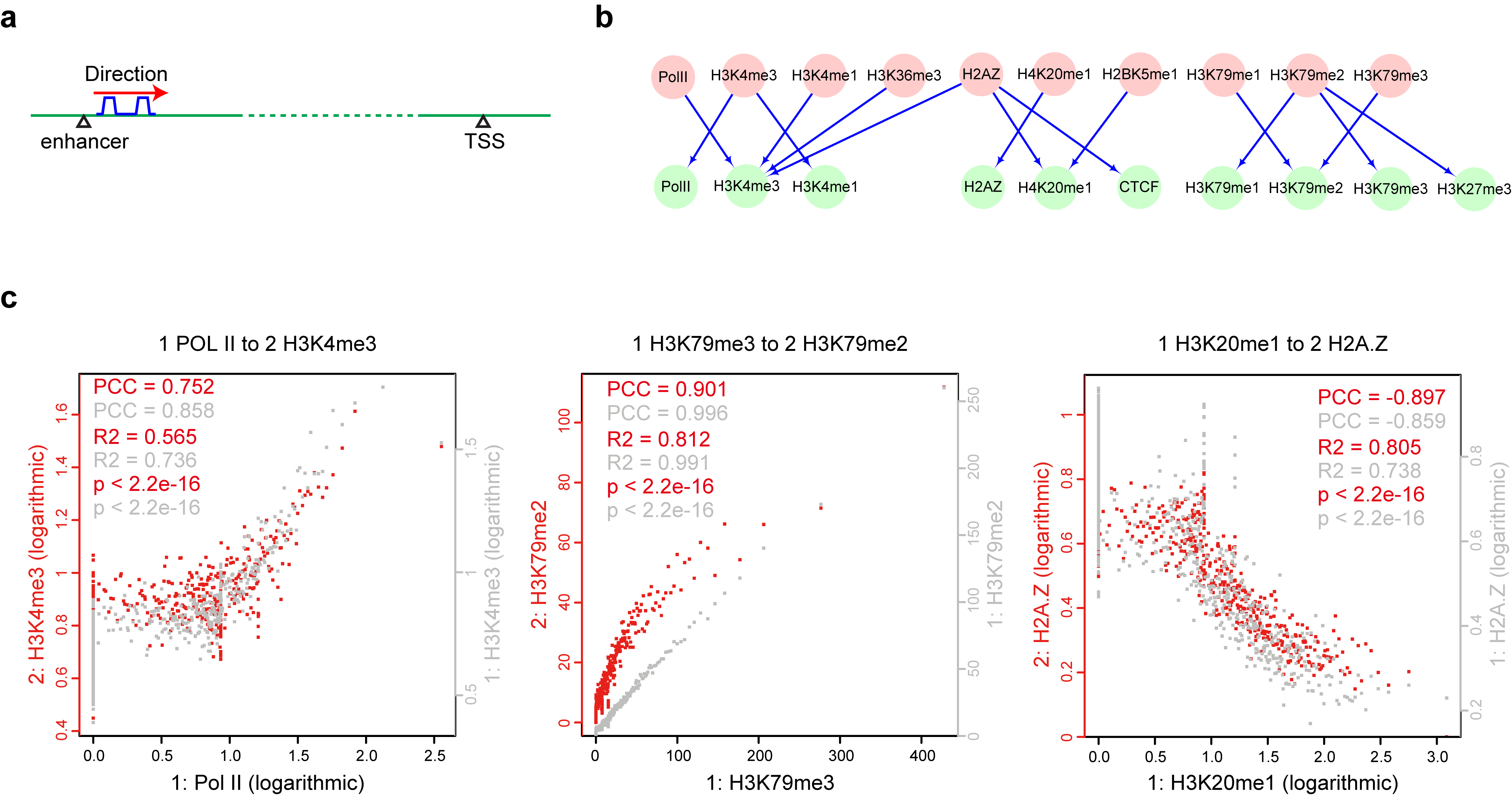

Supplement: S2 Fig — (a) Schematic diagrams of the direction of the modeled signal propagation at enhancer. (b) Consensus networks of inter-nucleosome propagation at enhancers. (c) Correlation between/among the factors in each of the three common modules. The analysis was the same as Fig 2, but between “+1” and “+2” nucleosomes at enhancer regions by the direction from the center of enhancer regions to the nearest TSSs. The correlation between factor A’s level at “before” nucleosomes and factor B’s level at “after” nucleosomes was illustrated by a scatter plot and quantified by Pearson correlation coefficient (PCC, red dots), while the on-site correlations (between the two different factors’ level at the same “before” nucleosomes) are shown with grey dots for comparison. (TIF) [file pcbi.1006416.s002.tif]

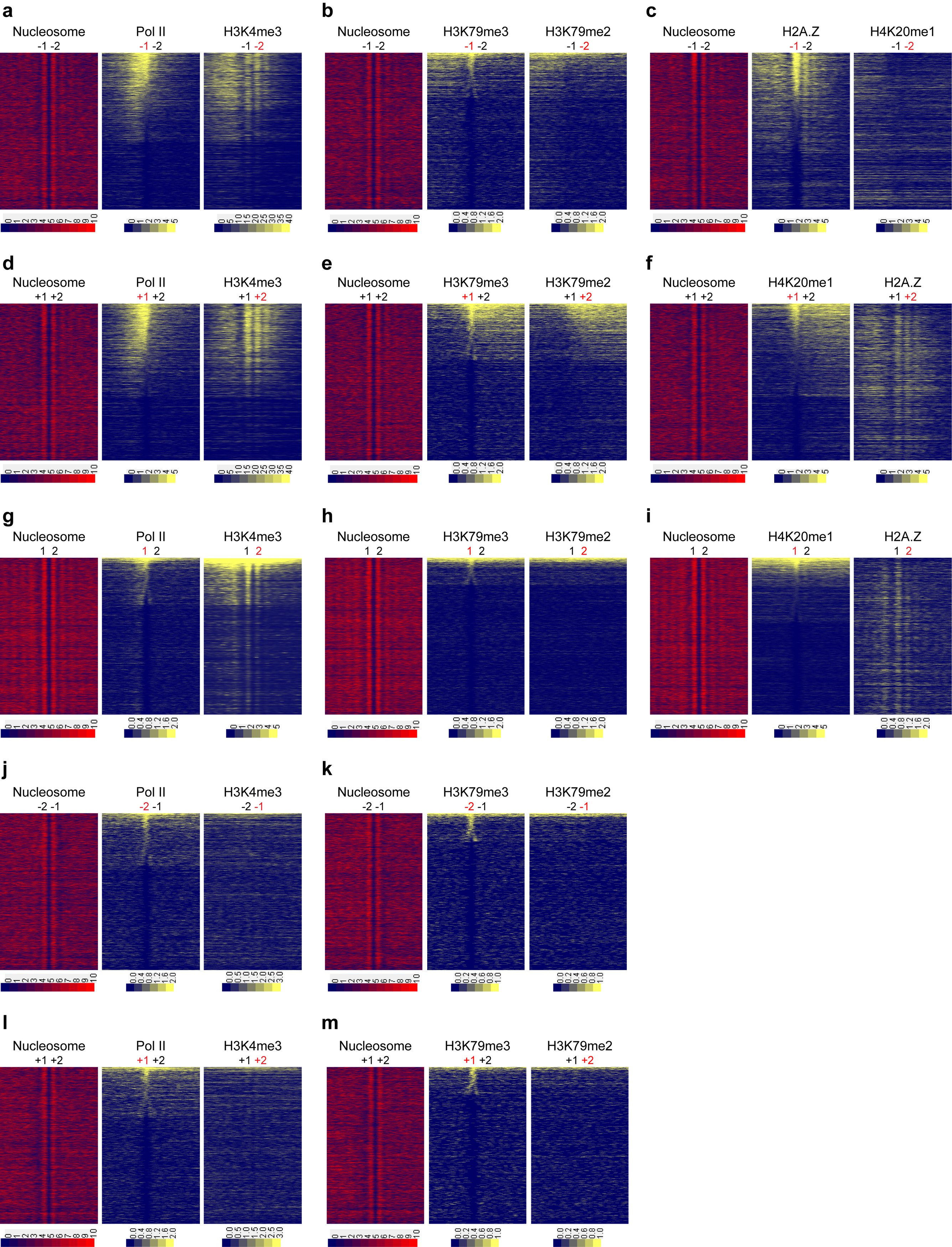

Supplement: S3 Fig — (a) Inter-nucleosome correlation for the propagation “Pol II → H3K4me3” from “-1” to “-2” nucleosomes at TSS regions by the direction from TSS center to upstream. The profiles of nucleosome, Pol II, and H3K4me3 signals were mapped to the -1000 to +1000 bp windows around each nucleosome pairs with a 10 bp resolution. The lines (or the corresponding TSSs) were ranked by Pol II signal of the “-1” nucleosome. (b-c) Same as (a), but for “H3K79me3 → H3K79me2” and “H2A.Z → H4K20me1” respectively. (d-f) Same as (a-c), but for the propagations from “+1” to “+2” nucleosomes at TSS regions by the direction from TSS center to downstream. (g-i) Same as (a-c), but from “1” to “2” nucleosomes around CTCF-binding regions in the direction from CTCF-binding sites to two flanking side. (f and i) Same as (c), but for the propagation “H4K20me1 → H2A.Z”. (j-k) Same as (a-b), but for the propagation from “-2” to “-1” nucleosomes at TTS regions by the direction from TTS upstream to TTS center. (l-m) Same as (a-b), but for the propagation from “+1” to “+2” nucleosomes at TTS regions by the direction from TTS center to TTS downstream. (TIF) [file pcbi.1006416.s003.tif]

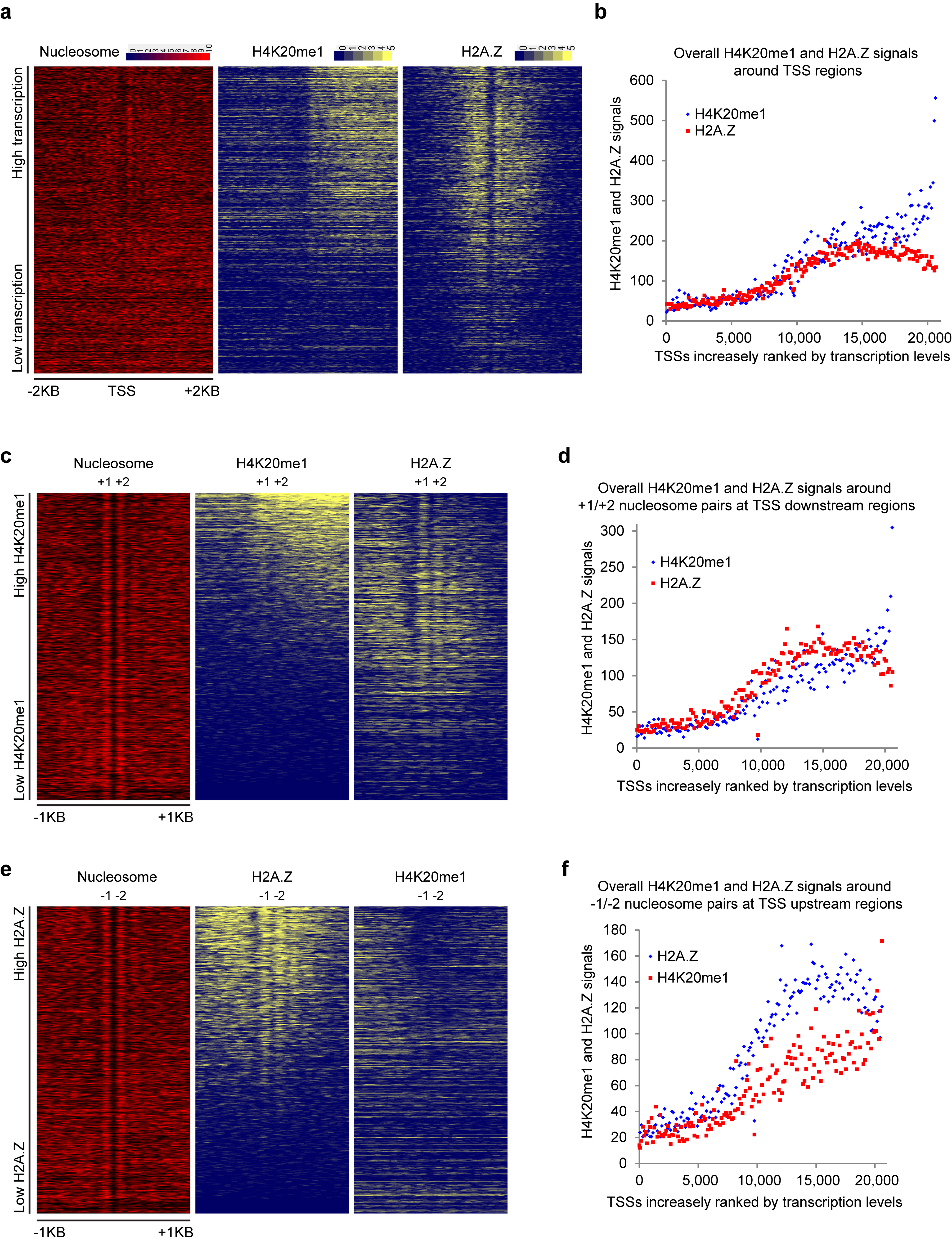

Supplement: S4 Fig — (a) H4K20me1 and H2A.Z signal profiles around TSSs. TSSs are decreasingly ranked by transcription levels. The profiles are in a 10 bp resolution within -2000 to +2000 bp around TSSs. (b) Cross-TSS correlation between H4K20me1 and H2A.Z. TSSs are increasingly ranked by transcription levels. Each point in the scatter plotting represents 100 TSSs grouped as a bin. The total H4K20me1 and H2A.Z signal intensities (after normalized by nucleosome signals) are quantified within -2000 to +2000 bp around TSS, and the mean value of each bin of 100 TSSs are shown. The cross-TSS Pearson correlation coefficient (PCC) is 0.811. (c-d) Similar as (a-b), but for the H4K20me1 and H2A.Z signals within -1000 to +1000 bp around the “+1/+2” nucleosome pairs of TSS downstream regions. The lines of heatmaps (c) are decreasingly ranked by the overall normalized H4K20me1 signals around each “+1/+2” nucleosome pair. PCC is 0.794 in (d). (e-f) Similar as (a-b), but for the H2A.Z and H4K20me1 signals within -1000 to +1000 bp around the “-1/-2” nucleosome pairs of TSS upstream regions. The lines of heatmaps (e) are decreasingly ranked by the overall normalized H2A.Z signals around each “-1/-2” nucleosome pair. PCC is 0.822 in (f). (TIF) [file pcbi.1006416.s004.tif]

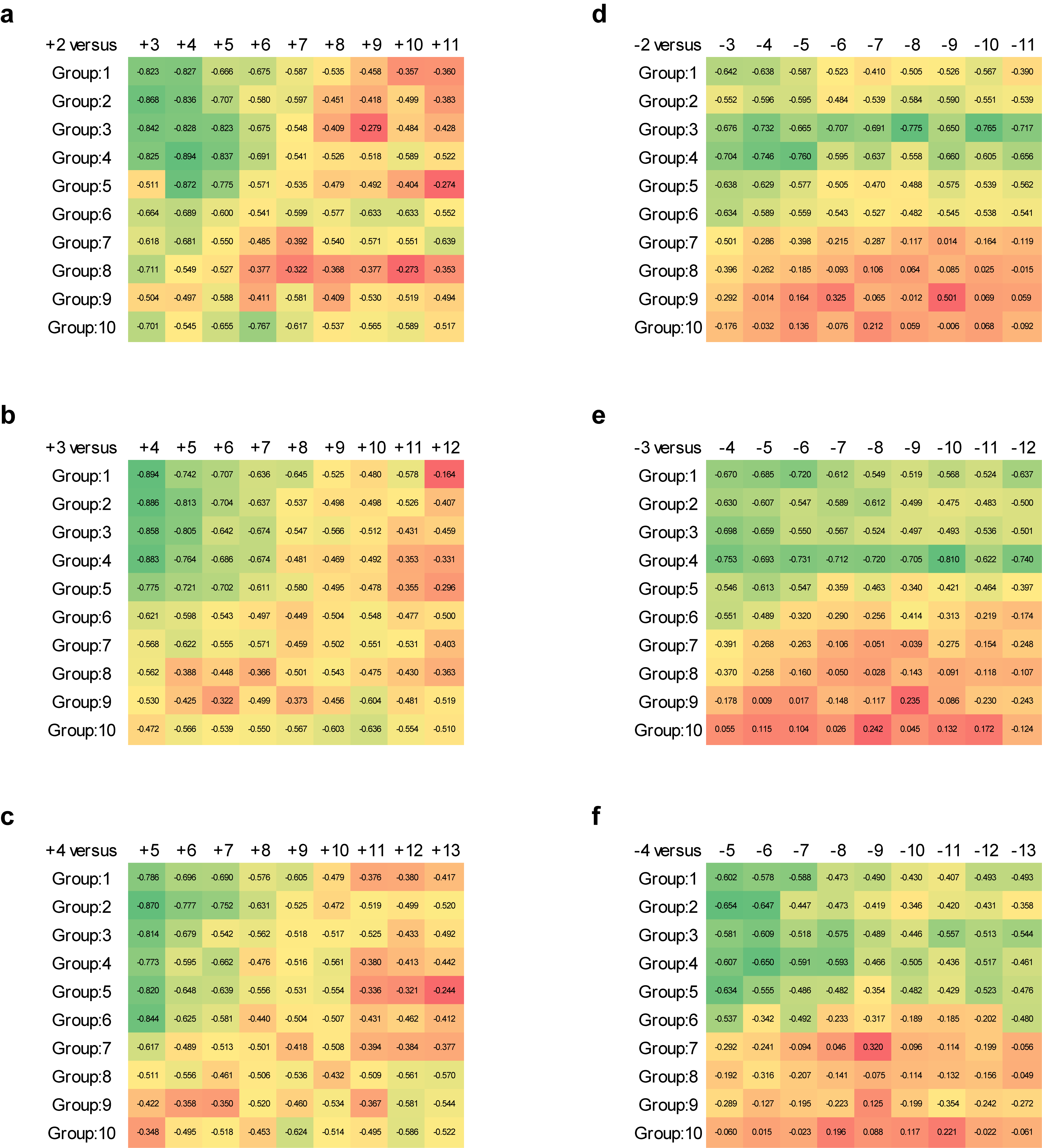

Supplement: S5 Fig — (a) H4K20me1/H2A.Z correlation at “+2” nucleosome versus surrounding nucleosomes along TSS downstream. Group 1~10 were obtained by decreasingly ranking TSSs according to transcription levels and evenly dividing them into 10 groups, as illustrated in Fig 3A. For each group, the cross-TSS Pearson correlation coefficient between “+2” and “+3”, “+4”, …, “+11” nucleosomes were calculated with each bin of 100 TSSs respectively. (b-c) Same as (a), but for “+3” (b) and “+4” (c) nucleosomes respectively. (d-f) Same as (a-c), but for H2A.Z/H4K20me1 correlation at “-2”, “-3” and “-4” nucleosome versus surrounding nucleosome along TSS upstream. (TIF) [file pcbi.1006416.s005.tif]

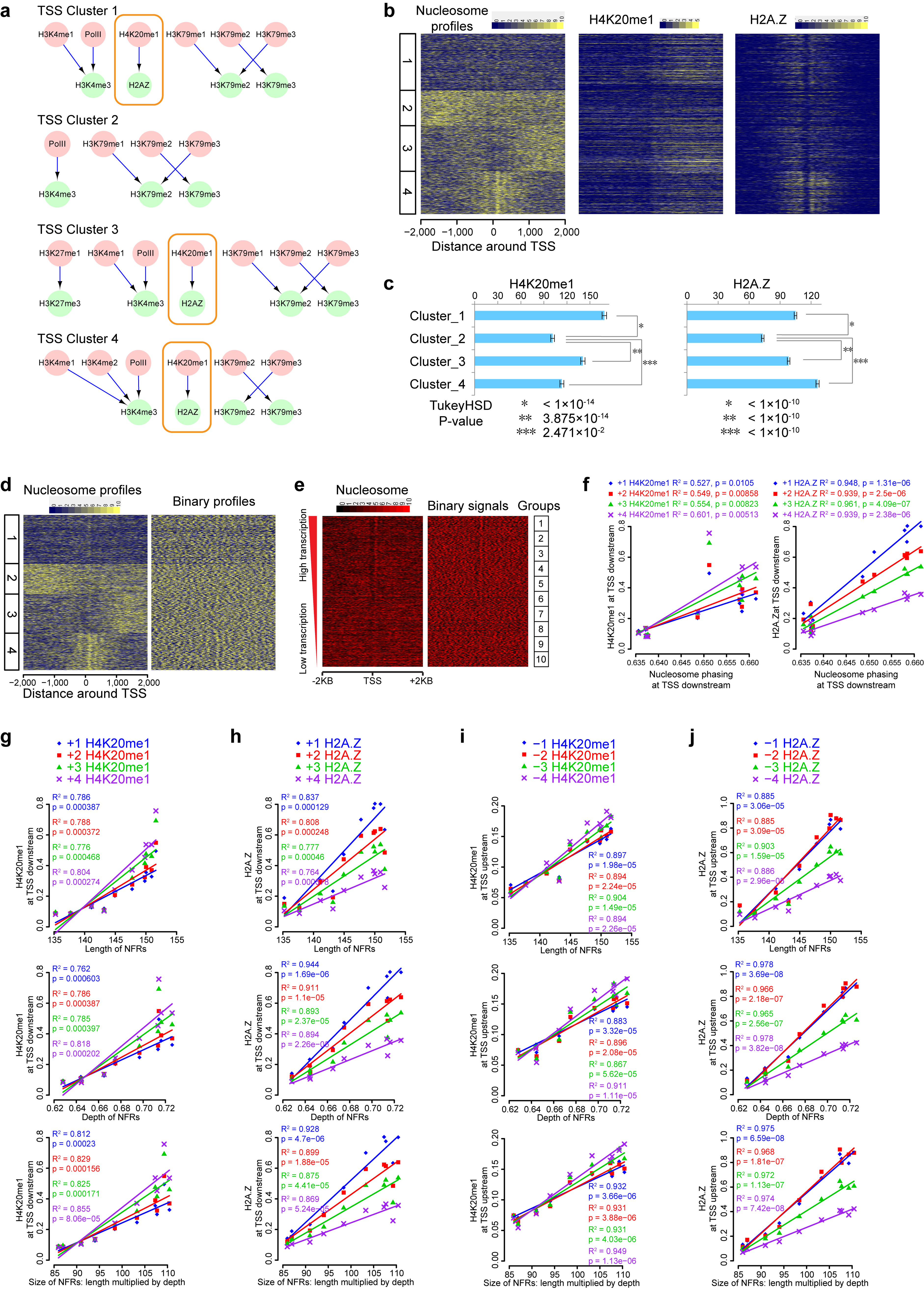

Supplement: S6 Fig — (a) The inter-nucleosome propagation network for each of the four clusters in Fig 4A. The DBN parameter “reg” was set to 2. The “H4K20me1 → H2A.Z” module is marked with box. (b) The H4K20me1/H2A.Z profiles within -2000 ~ +2000 bp regions surrounding TSS for the four nucleosome profile clusters shown in Fig 4A. (c) The total H2A.Z and H4K20me1 signal intensity (after normalized by nucleosome signals) within -2000 ~ +2000 bp regions surrounding TSS in each of the four clusters. The mean and standard error of the mean (SEM) are shown. One-way ANOVA was used for overall comparison, and TukeyHSD test was used to calculate the p-values between Cluster 2 and other clusters. (d) Binary nucleosome signals for the four TSS clusters. (e) Binary nucleosome signals for the TSSs decreasingly ranked by transcription levels. The TSSs are evenly divided into 10 groups. (f) Cross-group linear regression for H4K20me1 and H2A.Z levels (after normalized by nucleosome profiles) versus the strength of nucleosome phasing respectively. (g) Cross-group linear regression for H4K20me1 levels (after normalized by nucleosome profiles) at TSS downstream versus the length, depth, and size (length-multiplied-by-depth) of nucleosome free regions, respectively. Adjusted R2 and P-values are labeled on the panels. (h) Same as (g), but for H2A.Z at TSS downstream. (i-j) Same as (g-h), but for the H4K20me1 (i) and H2A.Z (j) intensities at TSS upstream. (TIF) [file pcbi.1006416.s006.tif]

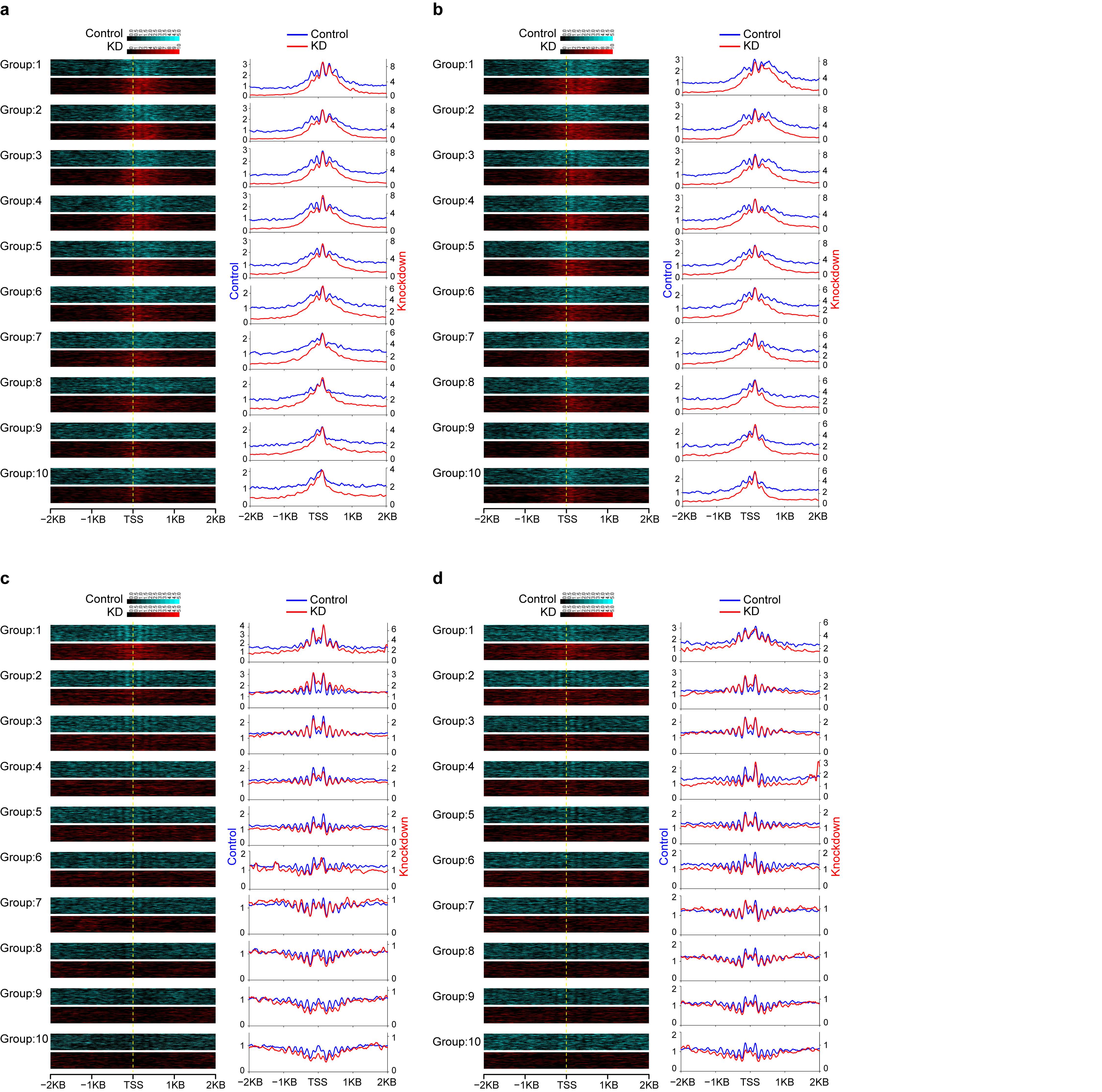

Supplement: S7 Fig — (a) Comparison of nucleosome profiles around TSSs between control and KD samples. Nucleosome profiles are aligned within -2000 to +2000 bp around TSSs in a 10 bp resolution. TSSs (having non-zero H2A.Z signals at “+2” nucleosomes) are decreasingly ranked by their H2A.Z levels at “+2” nucleosomes, and evenly divided into 10 groups. (b) Same as a, but based on the H2A.Z signals at “+3” nucleosomes. (c-d) Similar as a-b, but for the nucleosome profiles around CTCF-binding sites. (c) CTCF-binding sites (having non-zero H2A.Z signals at “1” nucleosomes) are decreasingly ranked by their H2A.Z levels at “1” nucleosomes, and evenly divided into 10 groups. (d) Same as c, but based on the H2A.Z signals at “2” nucleosomes. (TIF) [file pcbi.1006416.s007.tif]
