# Supplementary material for: Inter-nucleosomal communication between histone modifications for nucleosome phasing
Source: PLoS Comput Biol. 2018 Sep 6;14(9):e1006416. doi: 10.1371/journal.pcbi.1006416 (PMC6126837; doi:10.1371/journal.pcbi.1006416)
Supplement: S1 Text — (DOCX) [file pcbi.1006416.s008.docx]

**Supplementary Tables**

**Table A** DBN networks at various ranges around TSS/CTCF/TTS regions with different “center-inclusion levels”.

| **Region** | **center-**  **inclusion level** | **Range** | **Input pairs** | **Output edges** | **Area under ROC curve** |
| --- | --- | --- | --- | --- | --- |
| **TSS** | **Level 1** | **-1500 ~ +1500** |  |  |  |
|  |  | 0 ~ +1500 (TSS 🡪 downstream) | 18030 | 11 | 0.981 |
|  |  | -1500 ~ 0 (TSS 🡪 upstream) | 17896 | 7 | 0.989 |
|  |  | **-1750 ~ +1750** |  |  |  |
|  |  | 0 ~ +1750 (TSS 🡪 downstream) | 20898 | 9 | 0.972 |
|  |  | -1750 ~ 0 (TSS 🡪 upstream) | 20841 | 6 | 0.985 |
|  |  | **-2000 ~ +2000** |  |  |  |
|  |  | 0 ~ +2000 (TSS 🡪 downstream) | 23730 | 12 | 0.987 |
|  |  | -2000 ~ 0 (TSS 🡪 upstream) | 23727 | 10 | 0.983 |
|  | **Level 2** | **-1500 ~ +1500** |  |  |  |
|  |  | 0 ~ +1500 (TSS 🡪 downstream) | 18937 | 9 | 0.984 |
|  |  | -1500 ~ 0 (TSS 🡪 upstream) | 18811 | 5 | 0.984 |
|  |  | **-1750 ~ +1750** |  |  |  |
|  |  | 0 ~ +1750 (TSS 🡪 downstream) | 21796 | 9 | 0.978 |
|  |  | -1750 ~ 0 (TSS 🡪 upstream) | 21743 | 8 | 0.985 |
|  |  | **-2000 ~ +2000** |  |  |  |
|  |  | 0 ~ +2000 (TSS 🡪 downstream) | 24617 | 10 | 0.983 |
|  |  | -2000 ~ 0 (TSS 🡪 upstream) | 24619 | 7 | 0.980 |
|  | **Level 3** | **-1500 ~ +1500** |  |  |  |
|  |  | 0 ~ +1500 (TSS 🡪 downstream) | 19914 | 10 | 0.981 |
|  |  | -1500 ~ 0 (TSS 🡪 upstream) | 19983 | 8 | 0.970 |
|  |  | **-1750 ~ +1750** |  |  |  |
|  |  | 0 ~ +1750 (TSS 🡪 downstream) | 22763 | 12 | 0.992 |
|  |  | -1750 ~ 0 (TSS 🡪 upstream) | 22907 | 6 | 0.985 |
|  |  | **-2000 ~ +2000** |  |  |  |
|  |  | 0 ~ +2000 (TSS 🡪 downstream) | 25567 | 14 | 0.984 |
|  |  | -2000 ~ 0 (TSS 🡪 upstream) | 25770 | 9 | 0.986 |
| **CTCF** | **Level 1** | **-1000 ~ +1000** | 34129 | 14 | 0.975 |
|  |  | **-1500 ~ +1500** | 52052 | 22 | 0.995 |
|  |  | **-2000 ~ +2000** | 67873 | 28 | 0.992 |
|  | **Level 2** | **-1000 ~ +1000** | 36892 | 12 | 0.977 |
|  |  | **-1500 ~ +1500** | 54730 | 23 | 0.990 |
|  |  | **-2000 ~ +2000** | 70468 | 27 | 0.982 |
|  | **Level 3** | **-1000 ~ +1000** | 40460 | 14 | 0.977 |
|  |  | **-1500 ~ +1500** | 58201 | 23 | 0.984 |
|  |  | **-2000 ~ +2000** | 73854 | 28 | 0.973 |
| **TTS** | **Level 1** | **-1500 ~ +1500** |  |  |  |
|  |  | 0 ~ +1500 (TTS 🡪 downstream) | 15595 | 5 | 0.967 |
|  |  | -1500 ~ 0 (upstream 🡪 TTS) | 15792 | 6 | 0.970 |
|  |  | **-1750 ~ +1750** |  |  |  |
|  |  | 0 ~ +1750 (TTS 🡪 downstream) | 18455 | 6 | 0.979 |
|  |  | -1750 ~ 0 (upstream 🡪 TTS) | 18750 | 8 | 0.993 |
|  |  | **-2000 ~ +2000** |  |  |  |
|  |  | 0 ~ +2000 (TTS 🡪 downstream) | 21241 | 7 | 0.974 |
|  |  | -2000 ~ 0 (upstream 🡪 TTS) | 21619 | 11 | 0.979 |
|  | **Level 2** | **-1500 ~ +1500** |  |  |  |
|  |  | 0 ~ +1500 (TTS 🡪 downstream) | 16254 | 6 | 0.976 |
|  |  | -1500 ~ 0 (upstream 🡪 TTS) | 16466 | 6 | 0.985 |
|  |  | **-1750 ~ +1750** |  |  |  |
|  |  | 0 ~ +1750 (TTS 🡪 downstream) | 19106 | 9 | 0.975 |
|  |  | -1750 ~ 0 (upstream 🡪 TTS) | 19410 | 9 | 0.986 |
|  |  | **-2000 ~ +2000** |  |  |  |
|  |  | 0 ~ +2000 (TTS 🡪 downstream) | 21886 | 10 | 0.984 |
|  |  | -2000 ~ 0 (upstream 🡪 TTS) | 22274 | 11 | 0.991 |
|  | **Level 3** | **-1500 ~ +1500** |  |  |  |
|  |  | 0 ~ +1500 (TTS 🡪 downstream) | 17178 | 5 | 0.966 |
|  |  | -1500 ~ 0 (upstream 🡪 TTS) | 17673 | 6 | 0.991 |
|  |  | **-1750 ~ +1750** |  |  |  |
|  |  | 0 ~ +1750 (TTS 🡪 downstream) | 20016 | 7 | 0.967 |
|  |  | -1750 ~ 0 (upstream 🡪 TTS) | 20602 | 9 | 0.991 |
|  |  | **-2000 ~ +2000** |  |  |  |
|  |  | 0 ~ +2000 (TTS 🡪 downstream) | 22782 | 9 | 0.978 |
|  |  | -2000 ~ 0 (upstream 🡪 TTS) | 23439 | 12 | 0.992 |

The DBN parameter “reg” was set to 2.

**Table B** The synonyms of the 23 types of histone modifications/TFs used in co-citation analysis.

| **Terms** | **Terms with synonyms** |
| --- | --- |
| H3K4me1 | H3K4me1, H3K4 monomethylation, H3K4 mono-methylation, H3 Lys 4 monomethylation,  H3 Lys 4 mono-methylation, H3 lysine 4 monomethylation, H3 lysine 4 mono-methylation |
| H3K4me2 | H3K4me2, H3K4 dimethylation, H3K4 di-methylation, H3 Lys 4 dimethylation,  H3 Lys 4 di-methylation, H3 lysine 4 dimethylation, H3 lysine 4 di-methylation |
| H3K4me3 | H3K4me3, H3K4 trimethylation, H3K4 tri-methylation, H3 Lys 4 trimethylation,  H3 Lys 4 tri-methylation, H3 lysine 4 trimethylation, H3 lysine 4 tri-methylation |
| H3K9me1 | H3K9me1, H3K9 monomethylation, H3K9 mono-methylation, H3 Lys 9 monomethylation,  H3 Lys 9 mono-methylation, H3 lysine 9 monomethylation, H3 lysine 9 mono-methylation |
| H3K9me2 | H3K9me2, H3K9 dimethylation, H3K9 di-methylation, H3 Lys 9 dimethylation,  H3 Lys 9 di-methylation, H3 lysine 9 dimethylation, H3 lysine 9 di-methylation |
| H3K9me3 | H3K9me3, H3K9 trimethylation, H3K9 tri-methylation, H3 Lys 9 trimethylation,  H3 Lys 9 tri-methylation, H3 lysine 9 trimethylation, H3 lysine 9 tri-methylation |
| H3K27me1 | H3K27me1, H3K27 monomethylation, H3K27 mono-methylation, H3 Lys 27 monomethylation,  H3 Lys 27 mono-methylation, H3 lysine 27 monomethylation, H3 lysine 27 mono-methylation |
| H3K27me2 | H3K27me2, H3K27 dimethylation, H3K27 di-methylation, H3 Lys 27 dimethylation,  H3 Lys 27 di-methylation, H3 lysine 27 dimethylation , H3 lysine 27 di-methylation |
| H3K27me3 | H3K27me3, H3K27 trimethylation, H3K27 tri-methylation, H3 Lys 27 trimethylation,  H3 Lys 27 tri-methylation, H3 lysine 27 trimethylation, H3 lysine 27 tri-methylation |
| H3K36me1 | H3K36me1, H3K36 monomethylation, H3K36 mono-methylation, H3 Lys 36 monomethylation,  H3 Lys 36 mono-methylation, H3 lysine 36 monomethylation, H3 lysine 36 mono-methylation |
| H3K36me3 | H3K36me3, H3K36 trimethylation, H3K36 tri-methylation, H3 Lys 36 trimethylation,  H3 Lys 36 tri-methylation, H3 lysine 36 trimethylation, H3 lysine 36 tri-methylation |
| H3K79me1 | H3K79me1, H3K79 monomethylation, H3K79 mono-methylation, H3 Lys 79 monomethylation,  H3 Lys 79 mono-methylation, H3 lysine 79 monomethylation, H3 lysine 79 mono-methylation |
| H3K79me2 | H3K79me2, H3K79 dimethylation, H3K79 di-methylation, H3 Lys 79 dimethylation,  H3 Lys 79 di-methylation, H3 lysine 79 dimethylation, H3 lysine 79 di-methylation |
| H3K79me3 | H3K79me3, H3K79 trimethylation, H3K79 tri-methylation, H3 Lys 79 trimethylation,  H3 Lys 79 tri-methylation, H3 lysine 79 trimethylation, H3 lysine 79 tri-methylation |
| H3R2me1 | H3R2me1, H3R2 monomethylation, H3R2 mono-methylation, H3 Arg 2 monomethylation,  H3 Arg 2 mono-methylation, H3 arginine 2 monomethylation, H3 arginine 2 mono-methylation |
| H3R2me2 | H3R2me2, H3R2 dimethylation, H3R2 di-methylation, H3 Arg 2 dimethylation,  H3 Arg 2 di-methylation, H3 arginine 2 dimethylation, H3 arginine 2 di-methylation |
| H4K20me1 | H4K20me1, H4K20 monomethylation, H4K20 mono-methylation, H4 Lys 20 monomethylation,  H4 Lys 20 mono-methylation, H4 lysine 20 monomethylation, H4 lysine 20 mono-methylation |
| H4K20me3 | H4K20me3, H4K20 trimethylation, H4K20 tri-methylation, H4 Lys 20 trimethylation,  H4 Lys 20 tri-methylation, H4 lysine 20 trimethylation, H4 lysine 20 tri-methylation |
| H4R3me2 | H4R3me2, H4R3 dimethylation, H4R3 di-methylation, H4 Arg 3 dimethylation,  H4 Arg 3 di-methylation, H4 arginine 3 dimethylation, H4 arginine 3 di-methylation |
| H2BK5me1 | H2BK5me1, H2BK5 monomethylation, H2BK5 mono-methylation, H2B Lys 5 monomethylation,  H2B Lys 5 mono-methylation, H2B lysine 5 monomethylation, H2B lysine 5 mono-methylation |
| H2A.Z | H2AZ, H2A.Z |
| Pol II | RNA polymerase II, Pol II |
| CTCF | CCCTC binding factor , CCCTC-binding factor , CTCF |

**Table C** PubMed counting of pairwise co-citation for all the 23 histone modifications/TFs.

| H3K4me2 | 12 |  |  |  |  |  |  |  |  |  |  |  |  |  |  |  |  |  |  |  |  |  |
| --- | --- | --- | --- | --- | --- | --- | --- | --- | --- | --- | --- | --- | --- | --- | --- | --- | --- | --- | --- | --- | --- | --- |
| H3K4me3 | 18 | 42 |  |  |  |  |  |  |  |  |  |  |  |  |  |  |  |  |  |  |  |  |
| H3K9me1 | 0 | 2 | 4 |  |  |  |  |  |  |  |  |  |  |  |  |  |  |  |  |  |  |  |
| H3K9me2 | 4 | 26 | 25 | 8 |  |  |  |  |  |  |  |  |  |  |  |  |  |  |  |  |  |  |
| H3K9me3 | 1 | 13 | 36 | 6 | 30 |  |  |  |  |  |  |  |  |  |  |  |  |  |  |  |  |  |
| H3K27me1 | 0 | 1 | 1 | 1 | 2 | 1 |  |  |  |  |  |  |  |  |  |  |  |  |  |  |  |  |
| H3K27me2 | 0 | 4 | 6 | 1 | 4 | 1 | 4 |  |  |  |  |  |  |  |  |  |  |  |  |  |  |  |
| H3K27me3 | 3 | 28 | 106 | 4 | 33 | 50 | 6 | 13 |  |  |  |  |  |  |  |  |  |  |  |  |  |  |
| H3K36me1 | 0 | 0 | 0 | 0 | 0 | 0 | 0 | 0 | 0 |  |  |  |  |  |  |  |  |  |  |  |  |  |
| H3K36me3 | 1 | 4 | 24 | 0 | 6 | 13 | 1 | 0 | 13 | 0 |  |  |  |  |  |  |  |  |  |  |  |  |
| H3K79me1 | 0 | 0 | 0 | 0 | 0 | 0 | 0 | 0 | 0 | 0 | 0 |  |  |  |  |  |  |  |  |  |  |  |
| H3K79me2 | 1 | 1 | 9 | 1 | 0 | 1 | 0 | 0 | 2 | 0 | 3 | 1 |  |  |  |  |  |  |  |  |  |  |
| H3K79me3 | 0 | 1 | 2 | 0 | 0 | 1 | 0 | 0 | 1 | 0 | 3 | 1 | 3 |  |  |  |  |  |  |  |  |  |
| H3R2me1 | 0 | 0 | 0 | 0 | 0 | 0 | 0 | 0 | 0 | 0 | 0 | 0 | 0 | 0 |  |  |  |  |  |  |  |  |
| H3R2me2 | 0 | 1 | 3 | 0 | 0 | 1 | 0 | 0 | 1 | 0 | 0 | 0 | 0 | 0 | 0 |  |  |  |  |  |  |  |
| H4K20me1 | 0 | 2 | 3 | 2 | 2 | 2 | 1 | 1 | 2 | 0 | 1 | 0 | 0 | 0 | 0 | 0 |  |  |  |  |  |  |
| H4K20me3 | 0 | 4 | 8 | 0 | 5 | 20 | 0 | 1 | 13 | 0 | 1 | 0 | 1 | 0 | 0 | 0 | 4 |  |  |  |  |  |
| H4R3me2 | 0 | 0 | 0 | 0 | 0 | 1 | 0 | 0 | 0 | 0 | 0 | 0 | 0 | 0 | 0 | 0 | 0 | 1 |  |  |  |  |
| H2BK5me1 | 0 | 0 | 0 | 0 | 0 | 0 | 0 | 0 | 0 | 0 | 0 | 0 | 0 | 0 | 0 | 0 | 0 | 0 | 0 |  |  |  |
| H2A.Z | 0 | 1 | 6 | 0 | 2 | 1 | 0 | 0 | 2 | 0 | 0 | 0 | 0 | 0 | 0 | 0 | 0 | 1 | 1 | 0 |  |  |
| Pol II | 7 | 16 | 63 | 0 | 11 | 17 | 0 | 2 | 26 | 0 | 13 | 0 | 4 | 1 | 0 | 0 | 2 | 3 | 0 | 0 | 15 |  |
| CTCF | 1 | 2 | 6 | 0 | 1 | 10 | 0 | 0 | 10 | 0 | 2 | 1 | 1 | 1 | 0 | 0 | 0 | 2 | 0 | 0 | 3 | 25 |
|  | H3K4me1 | H3K4me2 | H3K4me3 | H3K9me1 | H3K9me2 | H3K9me3 | H3K27me1 | H3K27me2 | H3K27me3 | H3K36me1 | H3K36me3 | H3K79me1 | H3K79me2 | H3K79me3 | H3R2me1 | H3R2me2 | H4K20me1 | H4K20me3 | H4R3me2 | H2BK5me1 | H2A.Z | Pol II |

**Table D** DBN networks for different patterns of nucleosome profiles around TSS regions.

| **Region** | **Range** | **Input pairs** | **Output edges** | **Area under ROC curve** |
| --- | --- | --- | --- | --- |
| TSS cluster 1 | -2000 ~ +2000 | 13858 | 6 | 0.980 |
| TSS cluster 2 | -2000 ~ +2000 | 11842 | 4 | 0.987 |
| TSS cluster 3 | -2000 ~ +2000 | 15012 | 7 | 0.985 |
| TSS cluster 4 | -2000 ~ +2000 | 13713 | 6 | 0.983 |

Note that all the neighboring nucleosome pairs were selected at “center-inclusion level 1” (see Supplementary Figure S1a for the definition of “center-inclusion level”). The DBN parameter “reg” was set to 2.
